# Self‐Assembled Fluorescent Block Copolymer Micelles with Responsive Emission

**DOI:** 10.1002/anie.202117570

**Published:** 2022-03-04

**Authors:** Hannah Kurz, Christian Hils, Jana Timm, Gerald Hörner, Andreas Greiner, Roland Marschall, Holger Schmalz, Birgit Weber

**Affiliations:** ^1^ Department of Chemistry Inorganic Chemistry IV University of Bayreuth Universitätsstrasse 30 95447 Bayreuth Germany; ^2^ Macromolecular Chemistry and Bavarian Polymer Institute University of Bayreuth Universitätsstrasse 30 95440 Bayreuth Germany; ^3^ Department of Chemistry Physical Chemistry III University of Bayreuth Universitätsstrasse 30 95447 Bayreuth Germany

**Keywords:** Block Copolymers, Fluorescence, Micelles, Schiff Bases, Sensors

## Abstract

Responsive fluorescent materials offer a high potential for sensing and (bio‐)imaging applications. To investigate new concepts for such materials and to broaden their applicability, the previously reported non‐fluorescent zinc(II) complex **[Zn(L)]** that shows coordination‐induced turn‐on emission was encapsulated into a family of non‐fluorescent polystyrene‐block‐poly(4‐vinylpyridine) (PS‐b‐P4VP) diblock copolymer micelles leading to brightly emissive materials. Coordination‐induced turn‐on emission upon incorporation and ligation of the **[Zn(L)]** in the P4VP core outperform parent **[Zn(L)]** in pyridine solution with respect to lifetimes, quantum yields, and temperature resistance. The quantum yield can be easily tuned by tailoring the selectivity of the employed solvent or solvent mixture and, thus, the tendency of the PS‐b‐P4VP diblock copolymers to self‐assemble into micelles. A medium‐dependent off–on sensor upon micelle formation could be established by suppression of non‐micelle‐borne emission background pertinent to chloroform through controlled acidification indicating an additional pH‐dependent process.

## Graphical abstract

The encapsulation of Schiff base‐like zinc(II) complexes into micelles derived from polystyrene‐*block*‐poly(4‐vinylpyridine) diblock copolymers that offer coordination sites was investigated. Incorporation and ligation results in strongly emissive micelles with enhanced quantum yields and thermal stability compared to the neat complex in pyridine. The responsivity towards medium and acidity was utilized to design a *turn‐on* emission sensor material upon micelle formation.

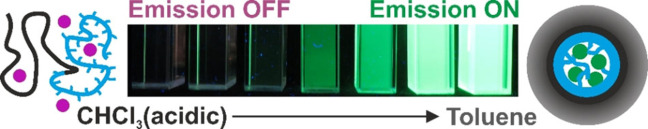

## Introduction

A very topical area of supramolecular research aims at tailor‐made responsive materials through encapsulation of functional units in nanocontainers such as coordination cages or micelles. Self‐assembled materials of this kind offer nanoscale confinements that are interesting for applications in the field of drug delivery,^[1]^ as reaction vessels,^[2]^ or storage rooms for reactive/instable molecules.^[3]^ Medium properties such as polarity will typically differ significantly between the insoluble, nanoscopic core of the micelles (confinement) and the surrounding medium (solvent and soluble micelle corona). If photoluminescent, nanocontainers can perform favorably as optical sensors, due to their high sensitivity towards the nature and properties of the nanoscopic environment, including viscosity or polarity, concentration‐dependent effects such as aggregation, and intermolecular interactions.[^4^, ^5^] In most reported cases, the nanocontainers are not directly involved in the diagnostic modulation of emission but offered a pseudo‐stationary, passive nanoconfinement for the incorporation of photoluminescent (multicomponent) compounds in a non‐luminescent matrix.^[6]^ For instance, Müller et al. reported on the determination of the critical micelle concentration (cmc) of block copolymer micelles, by using the fluorophore pyrene as a sensor for polarity changes upon micelle formation.[^5^, ^7^] Complementary approaches utilized fluorescent surfactants.^[8]^ Notably, both approaches are based on intrinsically fluorescent building blocks, in which the emissive properties can be modulated by external stimuli.

**Scheme 1:**
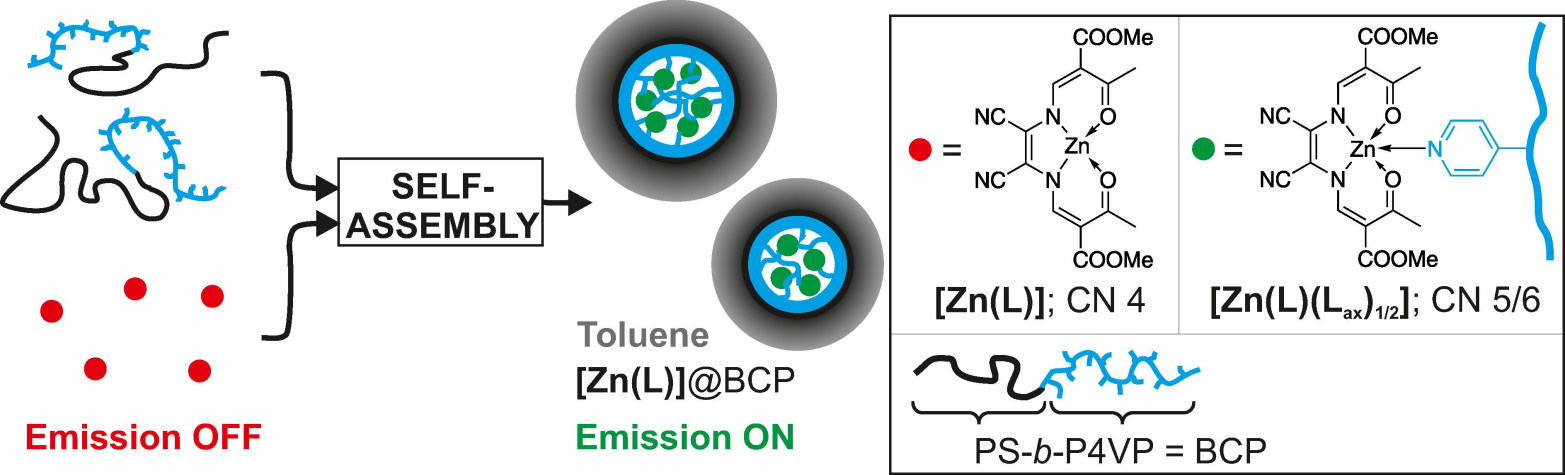
Representation of the basic principle of the self‐assembly of fluorescent micelles in toluene using the non‐fluorescent zinc(II) module **[Zn(L)]** with the used abbreviations. L: planar‐directing N_2_O_2_ ligand equipped with nitrile substituents.

In this study, we provide access to a new type of micelle sensor materials via a conceptually different approach. Herein, the block copolymer plays two roles, i) as the micelle building unit and ii) as the chemical stimulus to activate the emission of a latent fluorophore (see Scheme 1). Previous work from our labs has shown that polystyrene‐*block*‐poly(4‐vinylpyridine) (PS‐*b*‐P4VP) diblock copolymers can indeed act as a host for functional complexes through micellar self‐assembly in selective solvents such as THF and toluene.^[9]^ As Göbel et al. have demonstrated, the incorporation of switchable iron(II) spin crossover complexes or zinc(II) coordination polymers is driven by the coordination by the pendant pyridine units of the P4VP block.^[10]^ In this study, these pyridine moieties are used as anchoring units for sub‐coordinated zinc complexes. We adapt the effect of coordination‐activated emission of Schiff base zinc(II) complexes which had been put forward by Di Bella et al. Such zinc(II) complexes feature no emission in the stacked state in non‐coordinating solvents, whereas coordination‐induced destacking results in a *turn‐on* of a strong green emission.[^11^, ^12^]

Herein, we show that Di Bella's concept can indeed be transferred to micelles so that fluorescent micelles are synthesized via self‐assembly from non‐fluorescent hosts and non‐fluorescent guests. As shown in Scheme 1, we report on the synthesis and characterization of fluorescent micelles through encapsulation of the non‐fluorescent zinc(II) module **[Zn(L)]** into micelles derived from a family of non‐fluorescent PS‐*b*‐P4VP diblock copolymers. The encapsulated zinc(II) complex showed decent quantum yields of bright green fluorescence which tends to increase with the amount of anchoring sites in the micelle cores. Excited‐singlet state lifetimes indicate a massive stabilization of the emissive state against thermal deactivation upon encapsulation of **[Zn(L)]** into the micelles. Responsivity of emission was studied by varying solvent quality including the effect of medium‐responsive self‐assembly behavior and by addition of acide. Their potential as a thermally robust medium‐responsive fluorescent *turn‐on* sensor is highlighted.

## Results and Discussion

In selective non‐polar solvents such as toluene, micelles are formed through self‐assembly of the PS‐*b*‐P4VP BCPs. The low solubility of P4VP favors aggregation of the P4VP blocks to define the dense core of the micelles, whereas the soluble PS blocks form the corona with a significantly lower segment density.[^9^, ^13^] Empty micelles have been prepared from three different PS‐*b*‐P4VP BCPs with varying composition but comparable overall molecular weights (S_58_V_42_^157^, S_65_V_35_^131^, and S_85_V_15_^154^; subscripts: weight fraction of the respective block in wt %; superscript: number average molecular weight in kg mol^−1^). **[Zn(L)]**@BCP micelles were prepared by heating the respective BCP and **[Zn(L)]** (100 : 1 (w/w)) for 2 h under reflux in toluene. Afterwards, the solvent was removed and the compounds were dried in vacuo.

**Figure 1:**
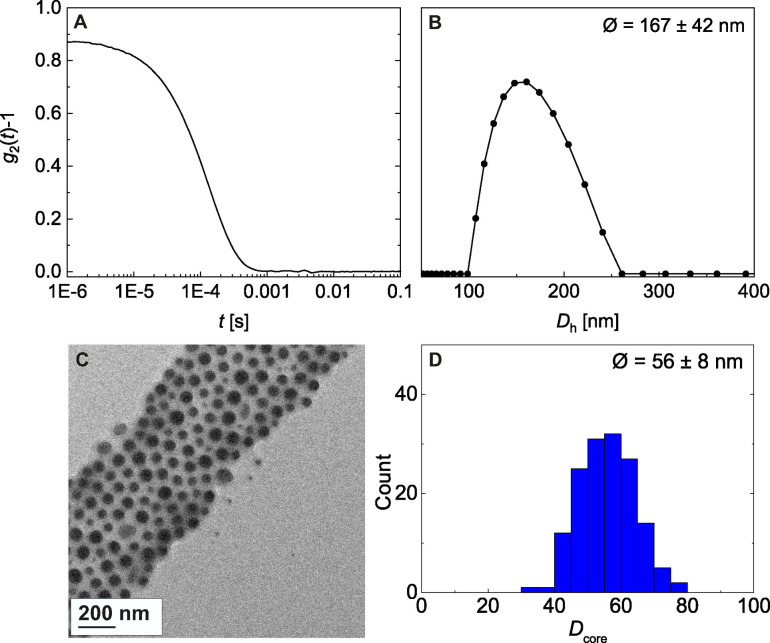
Autocorrelation function *g*_2_(*t*)‐1 vs. *t* (A) and *D*_h_ distribution of **[Zn(L)]**@S_58_V_42_^157^ micelles in toluene (B). TEM image of **[Zn(L)]**@S_58_V_42_^157^ micelles in toluene (C) and corresponding core size (*D*_core_) distribution derived from TEM image analysis (D). Due to the higher electron density contrast the Zn‐complex loaded P4VP cores of the micelles appear dark in TEM (C).

The hydrodynamic diameters (*D*_h_) and core diameters (*D*_core_) of the BCP micelles were determined by dynamic light scattering (DLS) and transmission electron microscopy (TEM), respectively (Table 1, Figure 1, Figure S1–S4). Hydrodynamic diameters *D*_h_ vary only slightly (150<*D*_h_<170 nm), irrespective of composition and/or complex load. By contrast, TEM measurements reveal a significant dependence of *D*_core_ on the weight fraction of the insoluble P4VP block for all micelles.

**Table 1:** *D*_h_ (DLS) and *D*_core_ (TEM) of the neat BCP and **[Zn(L)]**@BCP micelles in toluene.

Compound	*D*_h_ [nm]	*D*_core_ [nm]
S_58_V_42_^157^	170±46	64±7
**[Zn(L)]**@ S_58_V_42_^157^	167±42	56±8
S_65_V_35_^131^	151±39	51±6
**[Zn(L)]**@ S_65_V_35_^131^	152±37	47±6
S_85_V_15_^154^	159±40	30±5
**[Zn(L)]**@ S_85_V_15_^154^	160±44	31±4

For instance, the compound **[Zn(L)]**@S_85_V_15_^154^ with the lowest P4VP fraction features the smallest *D*_core_ (31±4 nm), whereas a nearly doubled *D*_core_ (56±8 nm) was observed for **[Zn(L)]**@S_58_V_42_^157^. Besides this size correlation, TEM measurements confirm that the incorporation of **[Zn(L)]** does not alter the morphology of the micelles as in all cases spherical micelles were obtained. DLS measurements show that all micelles were well‐dispersed in toluene and that the observed aggregation of the micelles in TEM images is due to drying effects upon sample preparation (Figure S1, S3).

**Figure 2:**
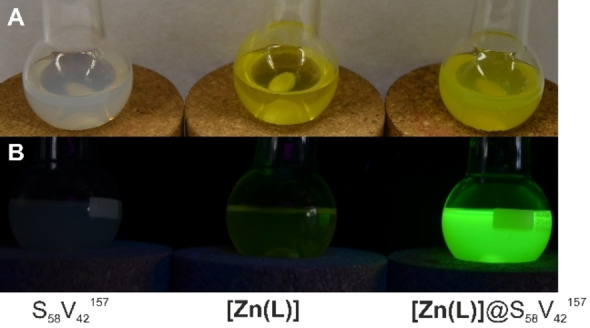
Photographs of the solutions from left to right of S_52_V_42_^157^ BCP (3.3 g L^−1^), **[Zn(L)]** (0.033 g L^−1^), and **[Zn(L)]**@S_58_V_42_^157^ (3.3 g L^−1^ BCP and 0.033 g L^−1^ **[Zn(L)]**) in toluene after heating under reflux for 10 min. Samples under day light (A) and in the dark under irradiation with *λ*_ex_=365 nm (B).

**Figure 3:**
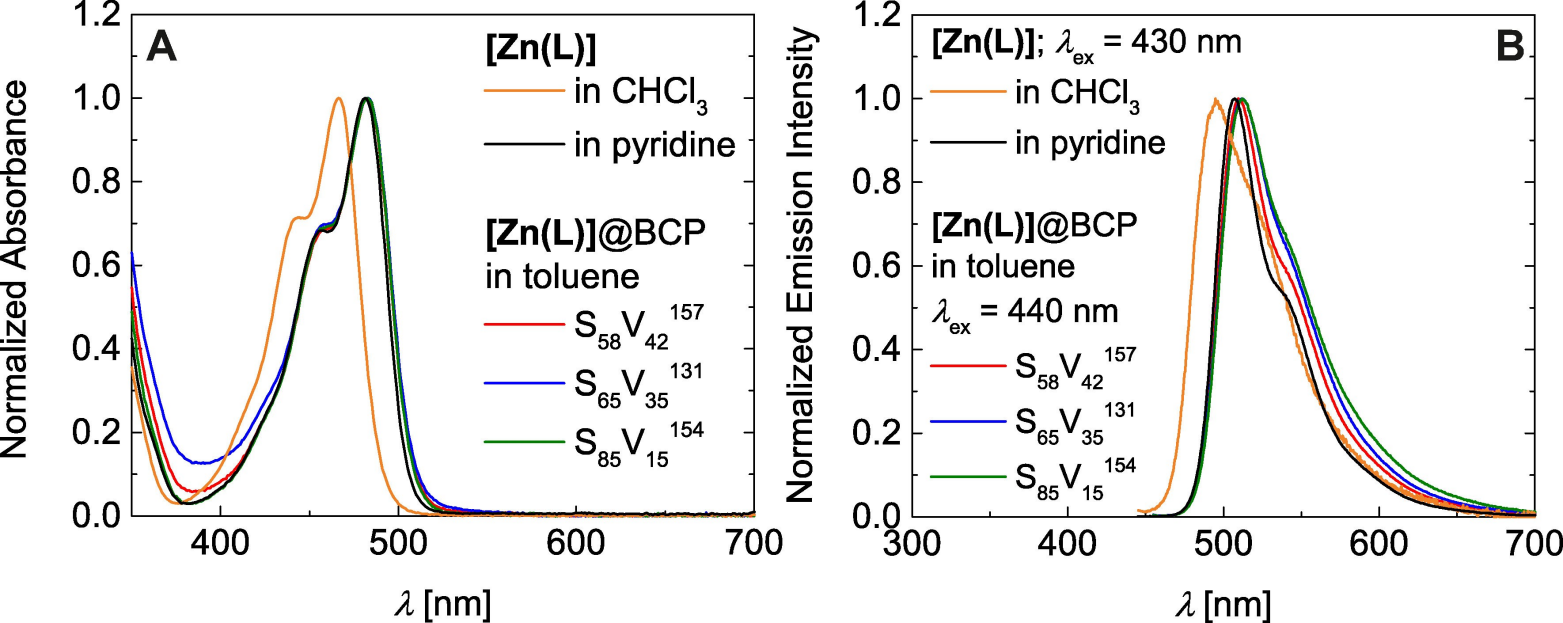
Normalized absorbance spectra of **[Zn(L)]**@BCP vs. neat BCP in toluene (A). Normalized emission spectra of **[Zn(L)]**@BCP in toluene (*λ*_ex_=440 nm, B).

Obviously, formation of **[Zn(L)]**@BCP hybrids in toluene results in a drastically enhanced emission. For instance, dispersions of micellar **[Zn(L)]**@S_58_V_42_^157^ feature a strong green emission (see Figure 2 right panel and video in the Supporting Information). By contrast, heating of either S_58_V_42_^157^ or **[Zn(L)]** separately in toluene does not result in an emissive solution (see Figure 2 left and middle panel and Figure S5B). The close‐to‐perfect match of both absorption and emission spectra of **[Zn(L)]**@BCP in toluene with the data of neat five‐coordinate **[Zn(L)py]** in pyridine, strongly hints towards incorporation of the initially non‐fluorescent **[Zn(L)]** into the micelle core and ligation of the pendant pyridine moieties of P4VP within the micelle core. All **[Zn(L)]**@BCP composites give absorption spectra, which nicely match the absorbance behavior of neat **[Zn(L)]** in pyridine (Figure 3A for normalized spectra and Figure S5C, S5D);^[12]^ namely two main absorption bands at *λ*=458 nm and *λ*=482 nm. Additionally, the corrected (for details please see Supporting Information) emission and fluorescence excitation spectra of the **[Zn(L)]**@BCP composites fully reflect the photoluminescent properties of **[Zn(L)]** in pyridine (Figure 3B for normalized spectra and Figure S5E, S5F).^[12]^ A strong green double‐peak emission with the emission maximum at *λ*_em,max_=509 nm, which can be clearly attributed to five‐coordinate **[Zn(L)x]**, is observed for all compounds (**x**: axial ligand). While the emission wavelength is similar for isoconcentrated samples (*c*=0.2 g L^−1^) of all three **[Zn(L)]**@BCP micelles, a remarkable difference in the emission intensity is observed (Figure S5E and photographs in Figure S6). Thereby, a higher P4VP content results in a higher emission intensity.

This finding is reflected as well in the quantum yields (*Φ*_Em_) and the excited state lifetimes (*τ*) of **[Zn(L)]**@BCP in toluene (Table 2). Hereby, **[Zn(L)]**@S_85_V_15_^154^ features the smallest quantum yield with 16 %, whereas a quantum yield of 33 % is observed for **[Zn(L)]**@S_58_V_42_^157^. The enhanced quantum yield goes along with longer lifetimes of the biexponential decays. This apparent correlation of the P4VP ratio and emission might be connected to the amount of potential donors. Indeed, simplified calculations reveal a significant divergence in the number of 4VP units relative to **[Zn(L)]** (ratios [4VP] : [Zn]=190 : 1 for S_58_V_42_^157^, 158 : 1 for S_65_V_35_^131^, and 68 : 1 for S_85_V_15_^154^; Table S1, detailed calculation is given in the Supporting Information). However, the general absence of the four‐coordinate species, as deduced from the absorbance spectra of all samples, contradicts this idea. Rather, the significant divergence in the excited state lifetimes may be referred to fluctuations through micelle dynamics that enables a higher degree of swelling. Clearly, non‐radiative deactivation is promoted in micelles with a smaller *D*_core_ that feature an increased surface to volume ratio. Moreover, the shorter P4VP block length enables a higher toluene content inside the core leading to increased dynamics.

**Table 2:** Quantum yields (*Φ*_E*m*_) and excited state lifetimes (*τ*) (with relative intensities) of **[Zn(L)]** in pyridine and **[Zn(L)]**@BCP in toluene derived from the lifetime measurements.

Compound	*Φ*_Em_ [%]	*τ*_1_ [ns] at 293 K	*τ*_2_ [ns] at 293 K
**[Zn(L)]** in pyridine	15	1.3 (100 %)	–
**[Zn(L)]**@S_58_V_42_^157^ in toluene	33	3.0 (62 %)	1.7 (38 %)
**[Zn(L)]**@S_65_V_35_^131^ in toluene	23	2.9 (51 %)	1.5 (49 %)
**[Zn(L)]**@S_85_V_15_^154^ in toluene	16	2.8 (34 %)	1.2 (66 %)

Interestingly, the quantum yields of all **[Zn(L)]**@BCP micelles (*Φ*_Em_=16–33 %) are higher than the quantum yield of neat **[Zn(L)]** in pyridine (*Φ*_Em_=15 %). This finding is also reflected in the longer excited state lifetimes of all **[Zn(L)]**@BCP composites in toluene compared to neat **[Zn(L)]** in pyridine (Table 2). Neat **[Zn(L)]** in pyridine features a monoexponential fluorescence decay with a lifetime of *τ*=1.3 ns at room temperature. By contrast, time‐correlated single photon counting (TCSPC) measurements of all **[Zn(L)]**@BCP composites gave biexponential decays with a longer lifetime of *τ*_1_=2.8–3.0 ns and a second shorter lifetime of *τ*_2_=1.2–1.7 ns.

**Figure 4:**
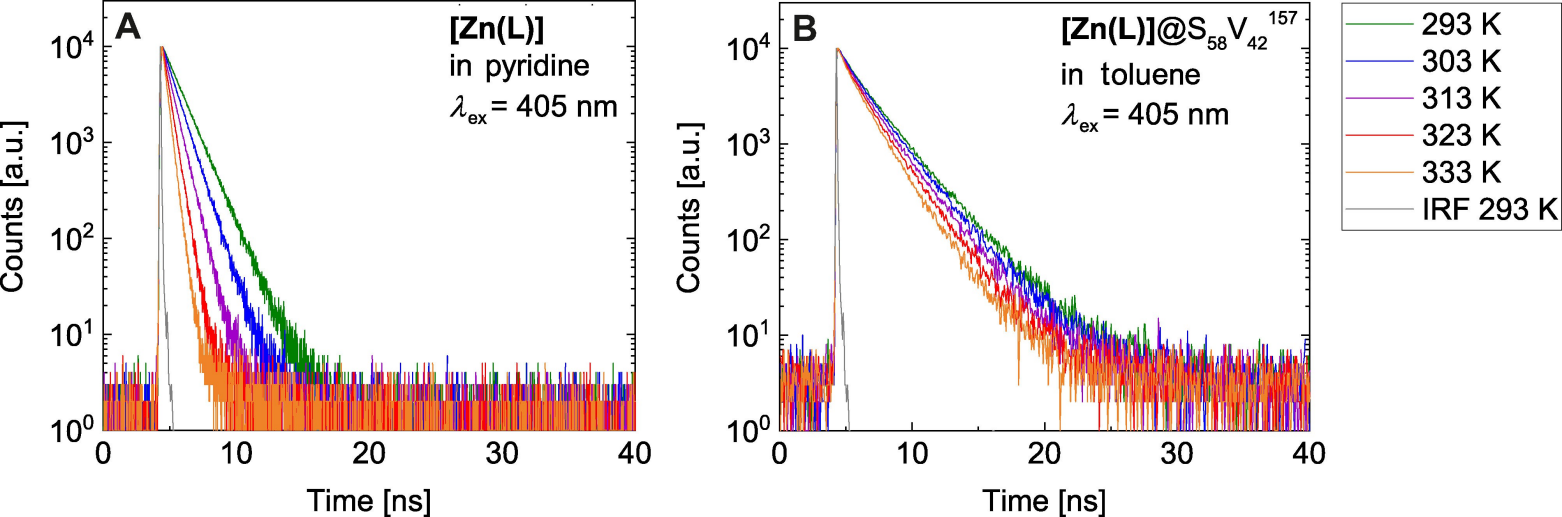
Temperature‐dependent fluorescence decays of **[Zn(L)]** in pyridine (A) and **[Zn(L)]**@S_58_V_42_^157^ micelles (B) in toluene (*λ*_ex_=405 nm; *λ*_em_=509 nm) detected by time‐correlated single photon counting (TCSPC).

Increased quantum yields of fluorophores due to embedment into micelles have been observed before.^[14]^ Typically, the more favorable photophysics is associated with the decreased mobility of the fluorophore inside the micelles, which disfavors non‐radiative deactivation. In the present case, this effect is even more impressive upon heating (Figure 4, Figure S7, S8 and Table S2). In contrast to the drastic lifetime decrease of neat **[Zn(L)]** in pyridine upon heating to 333 K (Arrhenius activation energy *E*_A_=26.1±1.1 kJ mol^−1^), **[Zn(L)]**@BCP show only a minor temperature dependence (**[Zn(L)]**@S_58_V_42_^157^: *E*_A_(*τ*_1_)=5.3±0.6 kJ mol^−1^, *E*_A_(*τ*_2_)=5.1±1.4 kJ mol^−1^, Figure S9). Strong changes of the viscosity can be ruled out within the measured temperature range, because even the highest temperature of 333 K is well below the glass transition of P4VP (*T*_g_≈420 K).^[15]^

The pyridine concentration dependency of the emission intensity of free **[Zn(L)]** was previously studied directly in chloroform/pyridine mixtures, where a *turn‐on* emission was observed upon addition of the coordinating solvent pyridine.^[12]^ As the **[Zn(L)]**@BCP composites show a remarkable thermally stable fluorescence in the micelle form, the question arose whether the micelle form is essential for the high emission and whether it is possible to form medium‐responsive sensor materials based on externally triggered self‐assembly induced emission.

**Scheme 2:**
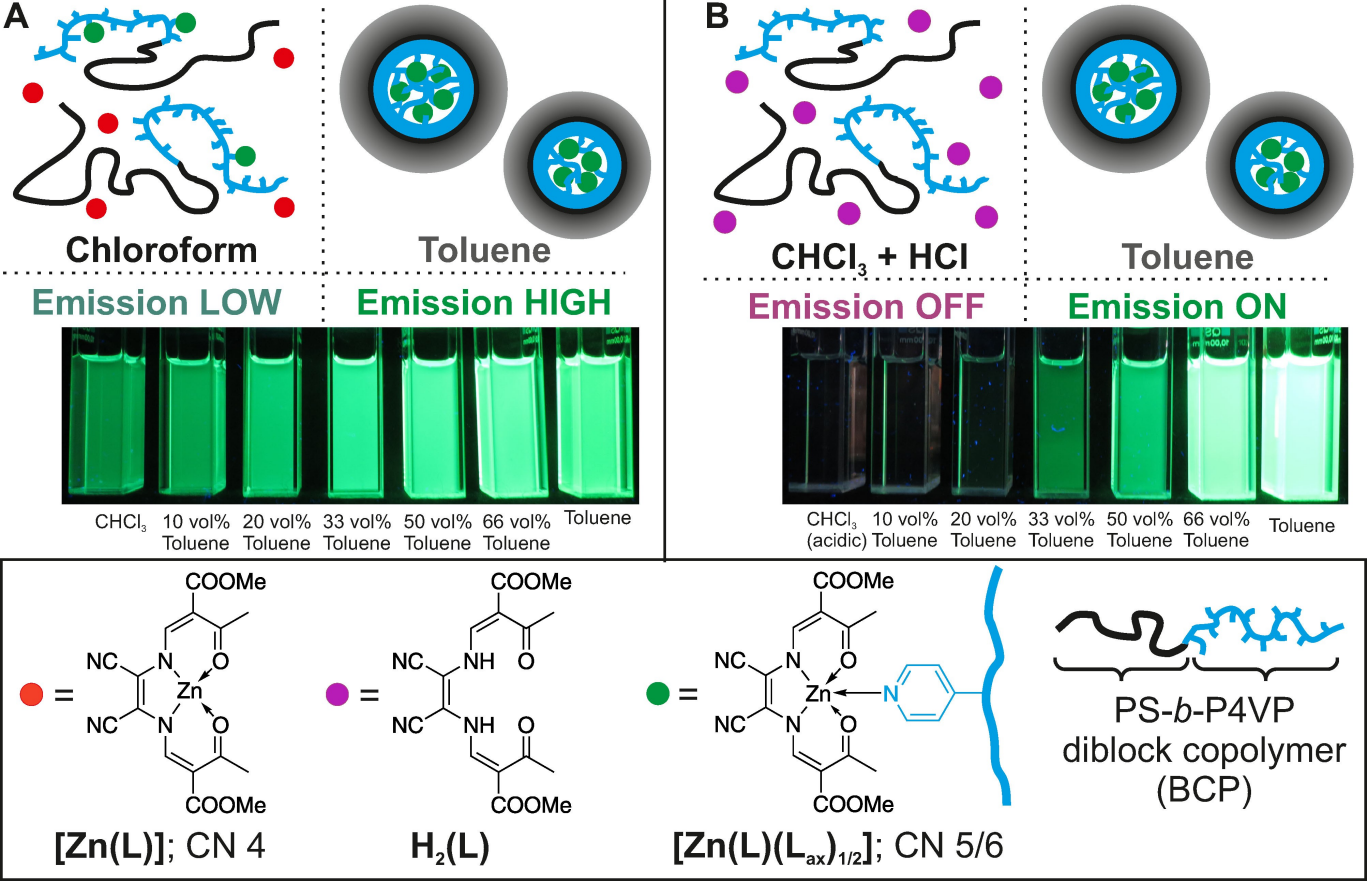
Representation of the basic principle of the medium‐dependent self‐assembly of an emission enhancement material based on BCP micelle formation in a CHCl_3_‐toluene series (A) and a CHCl_3_(acidic)–toluene series (B).

The impact of the micelle formation was studied in non‐coordinating solvent mixtures composed of a selective solvent for the PS block (toluene) inducing micelle formation and a non‐selective solvent, in which the BCP is molecularly dissolved in form of unimers (chloroform). Chloroform (CHCl_3_) is known as a non‐selective solvent for PS‐*b*‐P4VP diblock copolymers, allowing high solubility of both blocks.^[9]^ This results in molecularly dissolved PS‐*b*‐P4VP diblock copolymers (unimers) in toluene‐poor mixtures up to a threshold concentration of 20 vol % toluene (Figure S10, S11). The same observation holds for the composite **[Zn(L)]**@S_58_V_42_^157^, ruling out significant influence of the host on the micelle formation (Table 3, Figure S12, S13). Remarkably, the **[Zn(L)]**@BCP composites show a medium‐responsive emission behavior, making them highly interesting for sensor applications (Scheme 2).

**Table 3:** Hydrodynamic diameters: *D*_h_ (DLS) of the empty S_58_V_42_^157^ BCP micelles and **[Zn(L)]**@S_58_V_42_^157^.

	CHCl_3_–toluene series		CHCl_3_(acidic)– toluene series
	*D*_h_ [nm] of S_58_V_42_^157^	*D*_h_ [nm] of **[Zn(L)]**@S_58_V_42_^157^	*D*_h_ [nm] of **[Zn(L)]**@S_58_V_42_^157^
CHCl_3_	–^[a]^	–^[a]^	–^[a]^
10 vol % toluene	–^[a]^	–^[a]^	–^[a]^
20 vol % toluene	–^[a]^	–^[a]^	–^[a]^
33 vol % toluene	90±25	93±27	104±29
50 vol % toluene	114±34	120±37	131±32
66 vol % toluene	150±45	148±42	163±47
100 vol % toluene	156±34	161±38	160±37

[a] no micelle formation observed (Figure S10, S12, and S14).

Across an isoconcentrated series of **[Zn(L)]**@S_58_V_42_^157^ in CHCl_3_‐toluene mixtures, the emission clearly increases with increasing toluene content (Scheme 2A). Consequently, while the overall pyridine amount provided by the P4VP units of the BCP is not altered in the micelle formation process, the local effective concentration needs to be massively affected, when going from the molecularly dissolved form in CHCl_3_ to the micelle form in toluene. Indeed, the effective pyridine concentration is drastically lower in the molecularly dissolved form (5×10^−4^ M) compared to the micelle form (11 M) (calculations are given in the Supporting Information). Based on the optical properties of **[Zn(L)]**@S_58_V_42_^157^ in the CHCl_3_‐toluene series, we conclude that the emission changes are due the medium‐dependent self‐assembly of the micelles as shown in Scheme 2A.

Despite the low effective pyridine concentration in the molecularly dissolved form, still a non‐negligible amount of potentially coordinative pyridine units is present, which interferes with the aimed *off–on* behavior of the sensor. We reported previously on the close‐to‐zero emission of the ligand **H_2_(L)** in both solvents, CHCl_3_ and pyridine.^[12]^ To obtain a sharp *off–on* emission, we used acidified chloroform to consciously disrupt the parent zinc(II) complex to form the free ligand **H_2_(L)** and a soluble but not further defined zinc(II) species, according to: **[Zn(L)]**+2 H^+^→**H_2_(L)**+*Zn^2+^*.^[12]^ For this reason, CHCl_3_ was acidified by extraction with hydrochloric acid (see Experimental Section for details) and denoted as CHCl_3_(acidic) in the following. Upon micelle formation across the CHCl_3_(acidic)‐toluene series of **[Zn(L)]**@S_58_V_42_^157^, coordinated **[Zn(L)]** in the micelle core is increasingly protected from the acid by the basic pyridine substituents. This effect is further increased by the decreasing content of acid across the series and the lower accessibility of the core in toluene‐rich mixtures. This principle indeed allows *turn‐on* emission upon micelle formation as shown in Scheme 2B.

DLS measurements of the CHCl_3_‐toluene and CHCl_3_(acidic)‐toluene series show that the shape and size of the micelles are not significantly affected, neither by the presence of acid nor **[Zn(L)]**. DLS measurements of both series confirm that micelles are formed from a volume fraction of 33 vol % toluene onwards (*D*_h_ values in Table 3 and corresponding autocorrelation functions and *D*_h_ distributions in Figure S10–S15). A further rise of the toluene percentage results in an increase of *D*_h_ up to approximately 160 nm. While the presence of **[Zn(L)]** does not alter *D*_h_ at all, the values are slightly increased for the CHCl_3_(acidic)‐toluene mixtures. This finding indicates that the presence of acid results in enhanced swelling of the micelles.

Through extended absorbance, emission, and fluorescence excitation studies across both solvent series we could establish a direct correlation between the onset of micelle formation and on‐set of the emission rise. As the dynamics of block copolymer micelles are rather slow, we allowed all solutions to equilibrate for at least 30 min prior to measurements.^[16]^ As expected, the absorbance behavior of **[Zn(L)]**@S_58_V_42_^157^ in the non‐acidified CHCl_3_–toluene series is only marginally dependent on the CHCl_3_‐toluene mixing ratio. Only a minor red‐shift from *λ*=471 nm (CHCl_3_) to *λ*=483 nm (toluene) can be deduced (Figure S16A). This shift is in line with the observations made with free **[Zn(L)]** in CHCl_3_/pyridine mixtures at increased pyridine loads. It likewise indicates an enhanced coordination of **[Zn(L)]** at a higher toluene content.^[12]^ The slight absorbance changes observed even below 33 vol % toluene indicate some sort of pre‐aggregation prior to micelle formation, that is also reflected in the scattering of the neat BCP in the absorbance measurements even below micelle formation (Figure S17A). In the emission spectra, a successive emission intensity increase can be observed with increasing toluene content. Nevertheless, no *off–on* sensor behavior could be obtained due to a non‐negligible emission intensity observed for **[Zn(L)]**@S_58_V_42_^157^ in non‐acidified CHCl_3_ (see photograph in Scheme 2A and Figure S16B, D). Fluorescence excitation spectra confirm that this emission is based on axially coordinated **[Zn(L)]** (Figure S16C). Obviously, no medium‐dependent *turn‐on* sensor material was obtained due to a non‐negligible concentration of potentially coordinative P4VP units in CHCl_3_. However, the strongly increased effective pyridine concentration upon micelle formation and the stabilization of radiative pathways upon encapsulation result in a drastic emission enhancement.

Based on absorption spectra recorded of neat **[Zn(L)]** and encapsulated **[Zn(L)]**@S_58_V_42_^157^ across a CHCl_3_‐CHCl_3_(acidic) mixture series (Figure S18A, S19A and associated DLS profiles for **[Zn(L)]**@S_58_V_42_^157^ in Figure S20), we can attribute the acid‐dependent effects on the integrity of the zinc complex itself. The maximum absorbance band at *λ*=414 nm in acidified CHCl_3_ can be clearly attributed to the ligand **H_2_(L)**, which indicates (reversible) dissociation of the complex **[Zn(L)]**. Notably, acidification of CHCl_3_ results in a drastic emission reduction in both cases, **[Zn(L)]** and **[Zn(L)]**@S_58_V_42_^157^ (Figure S18B, S19B). The impact of acid was further verified by addition of acetic acid to a toluene solution of **[Zn(L)]**@S_58_V_42_^157^. Even addition of tiny amounts of organic acid results in the formation of the ligand and a drastic emission quenching, while conserving the micelle form (Figure S21, S22).

**Figure 5:**
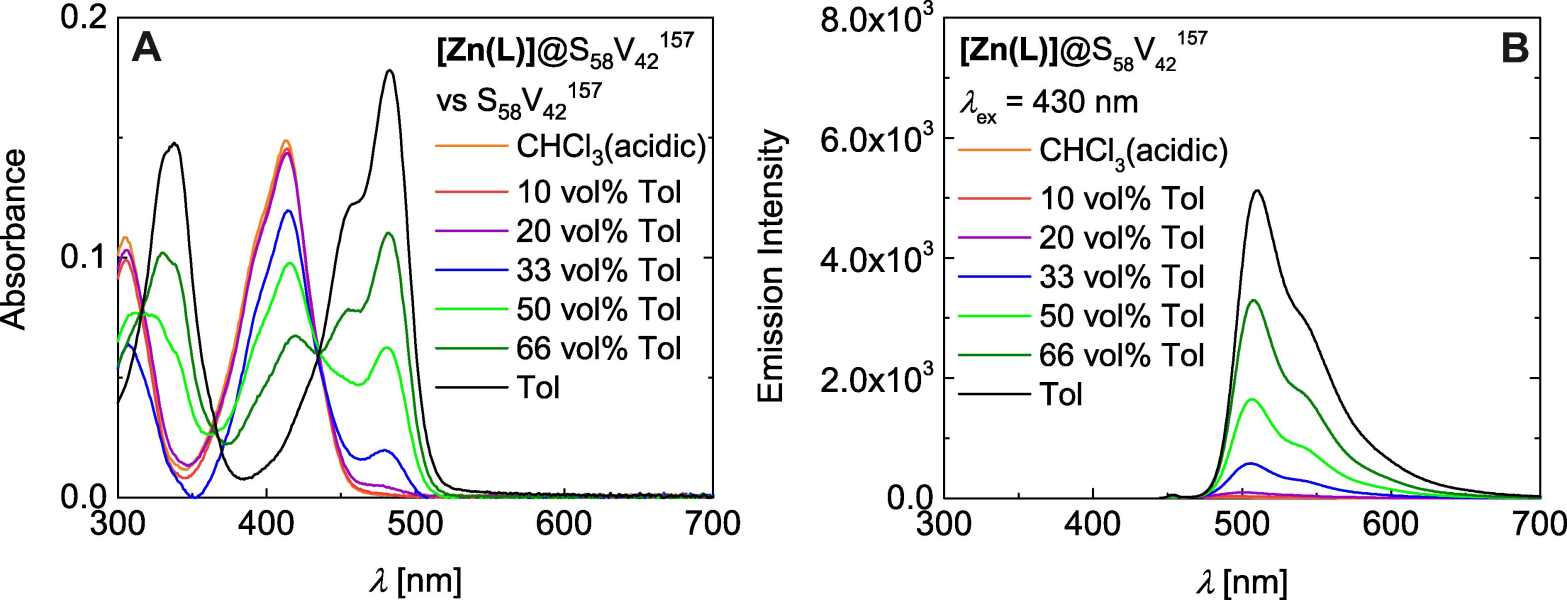
Absorbance (A) and emission (B) spectra of **[Zn(L)]**@S_58_V_42_^157^ in a CHCl_3_(acidic)–toluene series (*λ*_ex_=430 nm). Please note that the weak emission band at approximately *λ*=450 nm is a measurement artefact.

**Figure 6:**
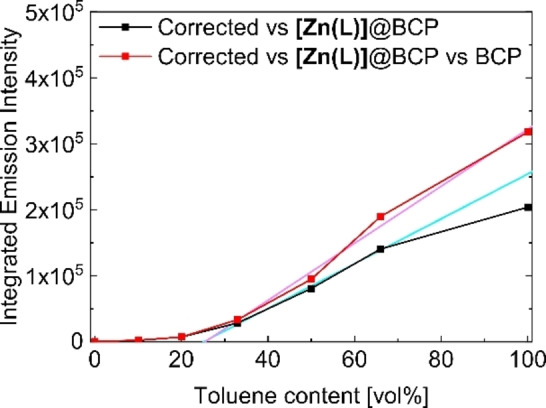
Plot of the integrated emission intensity vs. toluene content of **[Zn(L)]**@S_58_V_42_^157^. Two background correction possibilities are plotted to show that this trend is retained for both possibilities: Correction of the emission vs. the absorbance value at *λ*_ex_=430 nm i) of the pure solvent (red) and ii) of the empty BCP micelles (black). Please note that the emission intensity increase is slightly smaller when the scattering effects of the BCP micelles in the UV/Vis measurements are not taken into account (black curve). Hereby, the rather high scattering especially in pure toluene results in an artificially decreased emission. For this reason, this point was neglected in the linear fit (pink and cyan lines).

**Scheme 3:**
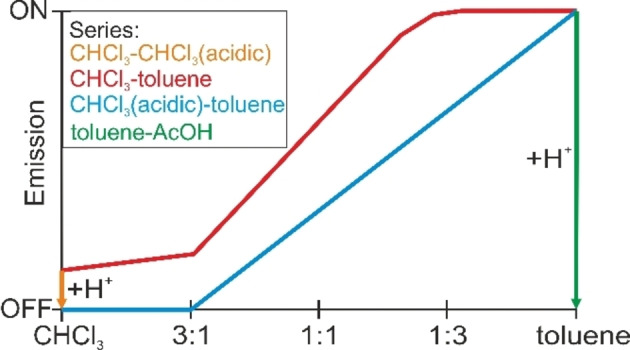
Summary of the medium‐dependent emission behavior of **[Zn(L)]**@S_58_V_42_^157^.

Remarkably, the absorption spectra of **[Zn(L)]**@S_58_V_42_^157^ remain indicative of the free ligand **H_2_(L)** up to a volume fraction of 20 vol % toluene in the series CHCl_3_(acidic)‐toluene (Figure 5A). Likewise, these solutions show no significant fluorescence, in accordance with the almost negligible emission of the ligand (*Φ*_Em_≈10^−4^).^[12]^ The normalized fluorescence excitation spectra confirm that the very weak emission is solely based on the ligand (normalized spectra in Figure S23). Only upon a further increase of the toluene content the specific absorbance band of **[Zn(L)]** at *λ*=479 nm re‐appears (Figure 5A). Comparison with the absorbance behavior of neat **[Zn(L)]** in pyridine indicates that the absorbance behavior in toluene‐rich mixtures is dominated by P4VP‐coordinated **[Zn(L)]** within the micelle core.^[12]^ The change in absorption is accompanied by a *switch‐on* emission (Figure 5B). The striking emission increase starting at 33 vol % toluene is shown in a photograph in Scheme 2B. Interestingly, the emission increases above 33 vol % toluene in a nearly linear manner (Figure 6). Hereby, two correction possibilities are plotted to show that this trend is retained for both possibilities: The correction of the emission versus the absorbance value at *λ*_ex_=430 nm using the pure solvent as background (red) and using the neat BCP micelles as background (black). Notably, both linear fits (pink and cyan lines) cross the x‐axis at approximately 25 vol % toluene. These values not only strongly support our finding that the micelle formation occurs in between 20 to 33 vol % as observed by DLS, but also that the micelle formation is the decisive requirement for efficient fluorescence. Thereby the micelles act as a unique host as they also provide the chemical stimulus for the *turn‐on* emission (Scheme 3).

## Conclusion

In this work PS‐*b*‐P4VP diblock copolymer (BCP) micelles with varying fractions of pyridine anchor groups are shown to be excellent, multifunctional platforms for the intra‐micellar fixation and activation of proto‐fluorophores. The family of **[Zn(L)]**@BCP materials, which anchor and activate non‐fluorescent **[Zn(L)]** within the micelles, exhibits a strong green emission with significantly enhanced quantum yields as compared to parent **[Zn(L)]** in pyridine. Embedding of the fluorophore within the insoluble P4VP core effectively prevents dynamic quenching, yielding longer fluorescent lifetimes, higher fluorescence quantum yields and very efficient protection against temperature variation. In combination with their facile processability, these favorable emission properties offer a wide applicability as strongly emissive pH‐responsive films. Embedding and activation of **[Zn(L)]** parallels the medium‐responsive self‐assembly of the diblock copolymer, yielding very weakly emissive aggregates in chloroform where the molecularly dissolved form is observed, whereas in toluene efficient micelle formation supports bright emission. According to DLS measurements, the micelle formation occurs in between 20 to 33 vol % toluene, which coincides with the onset of emission. An even sharper contrast between the emission of the molecularly dissolved form and the micelle form could be obtained through depression of the weak background in chloroform. Acidification to yield the close‐to‐zero emissive ligand **H_2_(L)** provides remarkable *turn‐on* emission that is coupled to the micelle formation in the acidified CHCl_3_(acidic)/toluene series of **[Zn(L)]**@S_58_V_42_^157^. Alternatively, the emission in toluene can be switched off upon addition of organic acids, while maintaining the micelle form. The basic principle to couple coordination‐induced emission with self‐assembled BCP micelles offers a new strategy to design fluorescent sensor materials for a broad application range.

## Conflict of interest

The authors declare no conflict of interest.

1

## Supporting information

As a service to our authors and readers, this journal provides supporting information supplied by the authors. Such materials are peer reviewed and may be re‐organized for online delivery, but are not copy‐edited or typeset. Technical support issues arising from supporting information (other than missing files) should be addressed to the authors.

Supporting Information

Supporting Information

## Data Availability

The data that support the findings of this study are available from the corresponding author upon reasonable request.
