# Supplementary material for: Self‐Assembled Fluorescent Block Copolymer Micelles with Responsive Emission
Source: Angew Chem Int Ed Engl. 2022 Mar 4;61(15):e202117570. doi: 10.1002/anie.202117570 (PMC9310857; doi:10.1002/anie.202117570)
Supplement: Supplementary file 1 — Supporting Information [file ANIE-61-0-s002.pdf]

Angewandte Chemie
Eine Zeitschrift der Gesellschaft Deutscher Chemiker

Supporting Information

Self-Assembled Fluorescent Block Copolymer Micelles with Responsive Emission

*H. Kurz, C. Hils, J. Timm, G. Hörner, A. Greiner, R. Marschall, H. Schmalz, B. Weber**

Experimental section

H_2(L) and **[Zn(L)]** were synthesized as described in literature.[1] Please note that the complex is only sparingly soluble in neat toluene even at a lower concentration of 0.025 g/L. The polystyrene-*block*-poly(4-vinylpyridine) (PS-*b*-P4VP) diblock copolymers (BCPs) were synthesized by sequential anionic polymerization of styrene and 4-vinylpyridine according to literature.[2] The nanocontainers are based on three different PS-*b*-P4VP-BCPs with varying composition but comparable overall molecular weights ($S_{58}V_{42}^{157}$, $S_{65}V_{35}^{131}$, and $S_{85}V_{15}^{154}$: subscripts give the fraction of the respective block in wt%; superscript denotes the number average molecular weight in kg mol^{-1}).[3] Toluene was of analytical grade and used without further purification. Chloroform was extracted with aqueous saturated $NaHCO_3$ solution and dried over $CaCl_2$. $CHCl_3$ (acidic) was prepared by extracting 50 mL dried $CHCl_3$ three times with 15 mL 4 M hydrochloric acid.

[Zn(L)]@BCP. Diblock copolymer $S_xV_y^z$ (0.150 g) and **[Zn(L)]** (0.0015 g) were heated in 60 mL toluene for 2 h under reflux. After cooling, the solvent was removed *in vacuo* and the yellow solid was dried *in vacuo*.

Characterization

Transmission electron microscopy (TEM) measurements were performed on a CEM902 microscope from Carl Zeiss Microscopy (Oberkochen, Germany). Samples were dispersed in toluene (c = 0.67 g L^{-1}) and the unfiltered solutions were dropped directly on a carbon coated TEM grid. The measurements were performed at an electron acceleration voltage of 80 kV. Micrographs were taken with an Orius 830 SC200W/DigitalMicrograph version 2.3 system from Gatan (Munich, Germany). Measurements were also performed on a Zeiss/LEO EM922Omega (Carl Zeiss Microscopy, Oberkochen, Germany) at an acceleration voltage of 160kV. Micrographs were taken with a CCD UltraScan camera system (Gatan, Munich, Germany) and acquiring software Digital Micrograph version 1.9 (Gatan, Munich, Germany). The software “ImageJ” developed by Wayne Rasband was used for the particles size determinations.[4] The diameter of 150 particles was determined and averaged.

Dynamic light scattering (DLS) measurements were conducted on an AntonPaar Litesizer 500 in fluorescence quartz glass cuvettes with a 1 cm light pathway from Hellma. The measurements were performed in backscattering mode and consisted of

six consecutive runs. The samples were dispersed in toluene (c = 0.2 g L^{-1}) and were not filtered. The experimental data were fitted with a cumulative fit. Measurements were also performed on an ALV DLS/SLS-SP 5022F compact goniometer system with an ALV 5000/E cross-correlator at a scattering angle of q = 90° and at T = 23 °C, using a HeNe laser (max. 35 mW, l = 632.8 nm) as the light source. The time-dependent scattering intensity was monitored with an APD (avalanche photodiode)-based pseudo cross correlation system. All samples were filled into NMR tubes (VWR, 5 mm outer diameter) for measurement. For each sample at least 3 measurements were performed. The data were evaluated using ALV Correlator software (version V.3.0.0.17 10/2002) and the implemented ALV regularized fit option ($g2(t)$, CONTIN-analysis).

Absorption spectra were performed on a Cary 60 UV-Vis spectrometer from Agilent Technologies. The samples were dispersed in toluene (c = 0.2 g L^{-1}) and were not filtered. For **steady-state photoluminescence** (PL) a FP-8600 fluorescence spectrometer from JASCO was employed that is equipped with a 150 W Xe lamp as excitation source. **Time-correlated single photon counting** (TCSPC) measurements to determine emission lifetimes were performed on a FluoTime 300 spectrofluorometer from PicoQuant, using a 405 nm diode laser for excitation (Coherent COMPASS 405-50 CW), which was controlled by the PDL 820 PicoQuant laser driver. **Quantum yields** were determined at room temperature using a 78mm integrating sphere and a 300 W Xe lamp as excitation source. All measurements were conducted in quartz cells with a 1 cm lightpath from Hellma.

<u>Correction of the optical data</u>

Neat BCP in the respective solvent mixture was used as background for the **[Zn(L)]**@BCP samples in the absorbance measurements to subtract the contributions of the neat BCP micelles on the absorption behavior (see Figure S5A/C/D and S15A in the SI). Please note, that the emission data was corrected against the absorbance at the excitation energy.

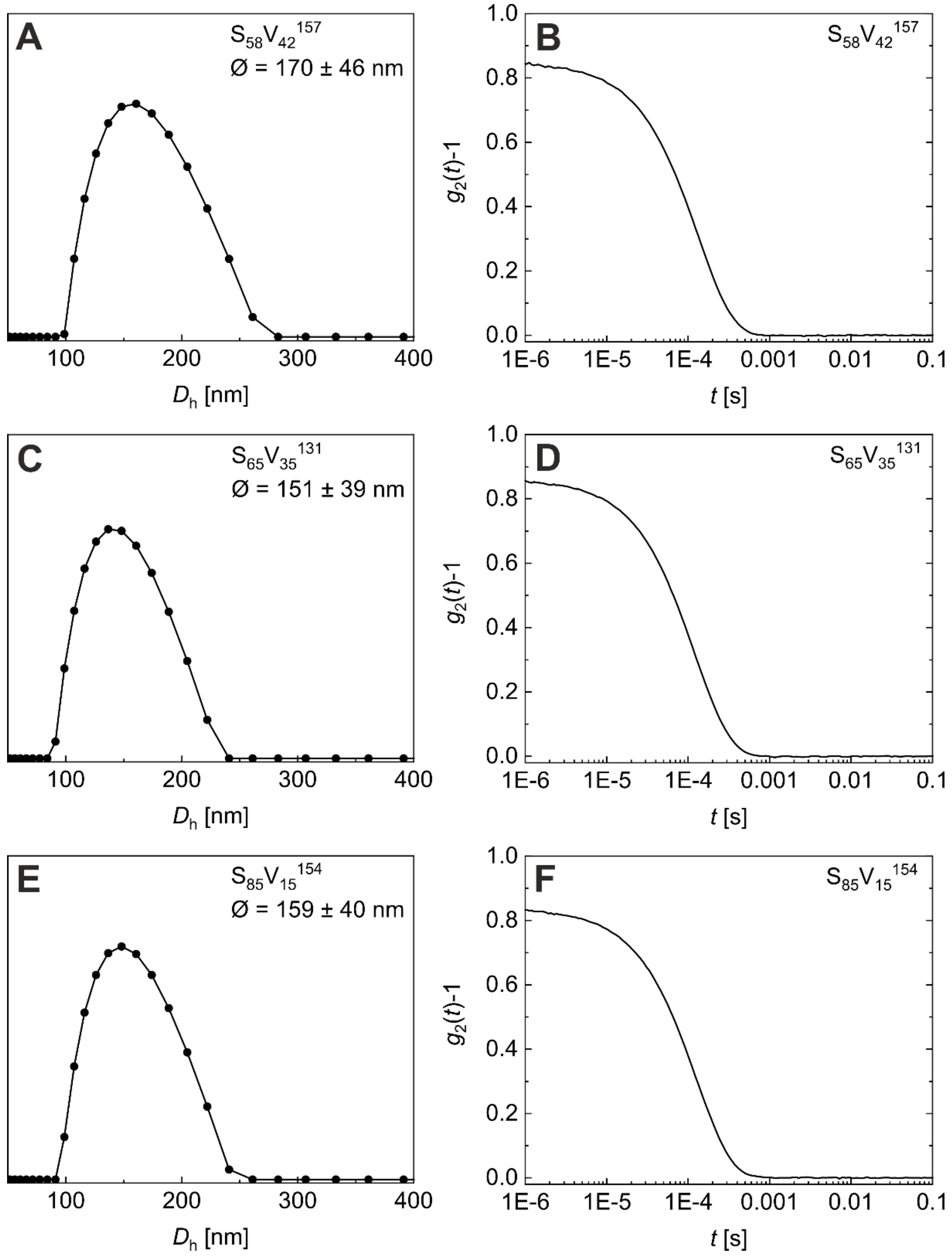

Figure S1: DLS measurements of neat BCP micelles in toluene: Hydrodynamic diameter (D_h) distributions and corresponding autocorrelation functions $g_2(t)$-1 vs t of $S_{58}V_{42}^{157}$ (A, B), $S_{65}V_{35}^{131}$ (C, D), and $S_{85}V_{15}^{154}$ (E, F).

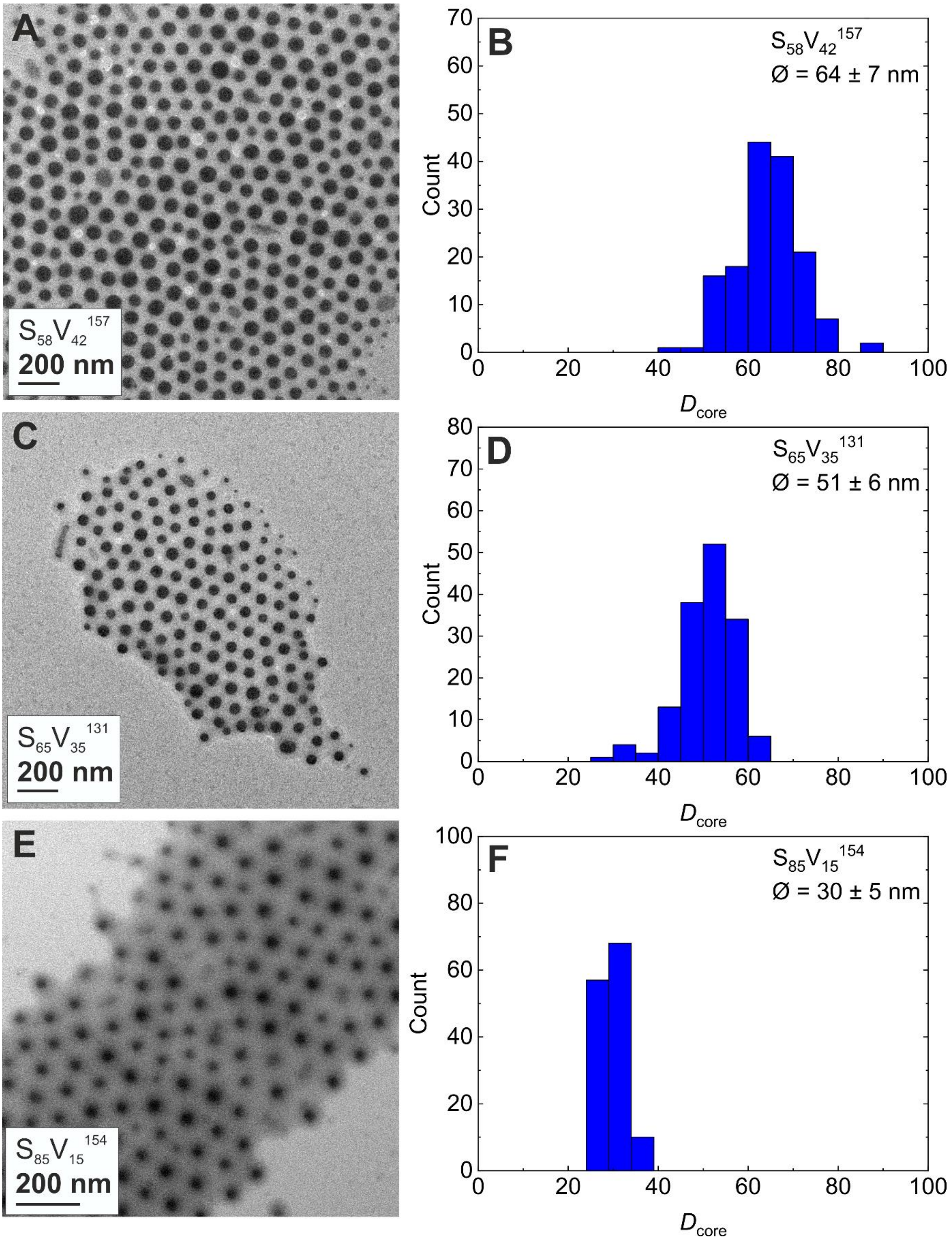

Figure S2: TEM images of neat $S_{58}V_{42}^{157}$ (A), $S_{65}V_{35}^{131}$ (C), and $S_{85}V_{15}^{154}$ (E) micelles in toluene and corresponding core size (D_{core}) distributions derived from TEM image analysis (B, D, F). Please note the different scale in E. The micelle core can be observed in TEM measurements due to the higher electron density contrast of the dense P4VP block compared to the PS corona.

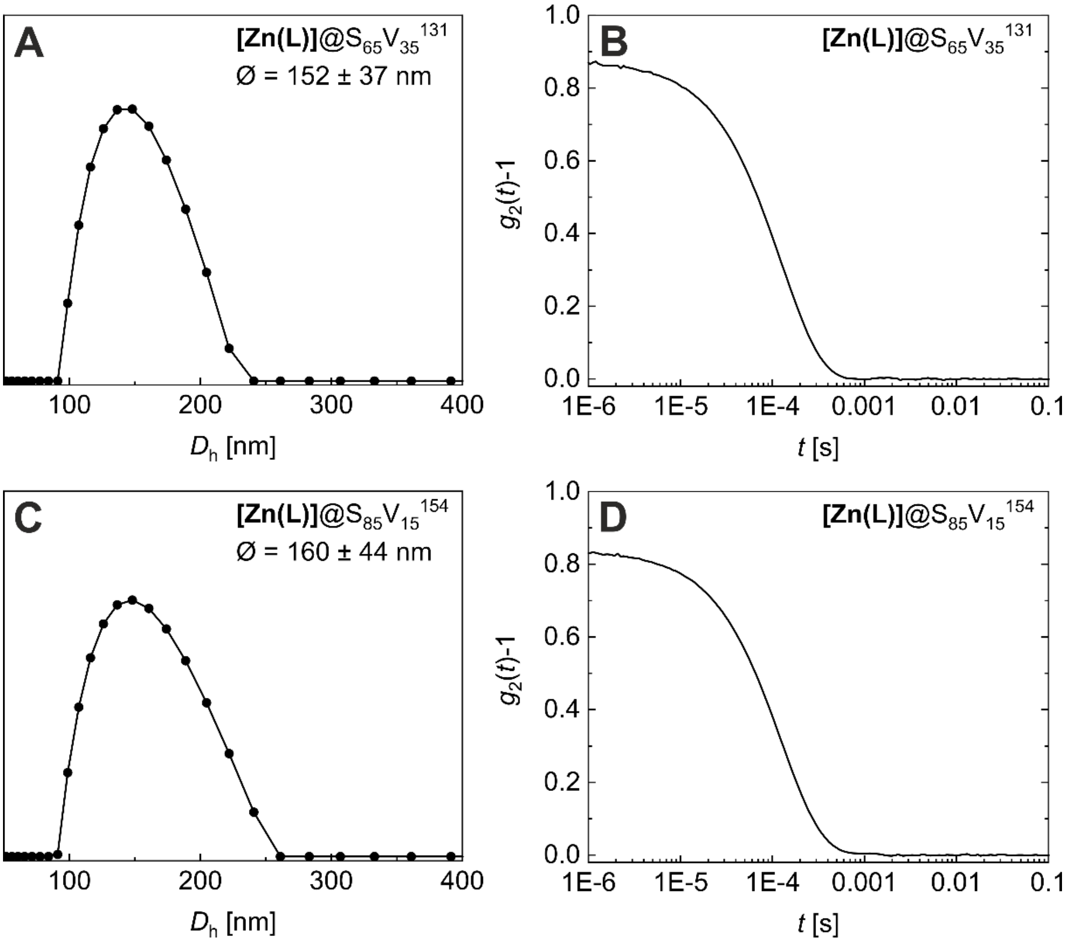

Figure S3: DLS measurements of **[Zn(L)]**@BCP in toluene: Hydrodynamic diameter (D_h) distributions and corresponding autocorrelation functions $g_2(t)$-1 vs t of **[Zn(L)]**@$S_{65}V_{35}^{131}$ (A, B) and **[Zn(L)]**@$S_{85}V_{15}^{154}$ (C, D).

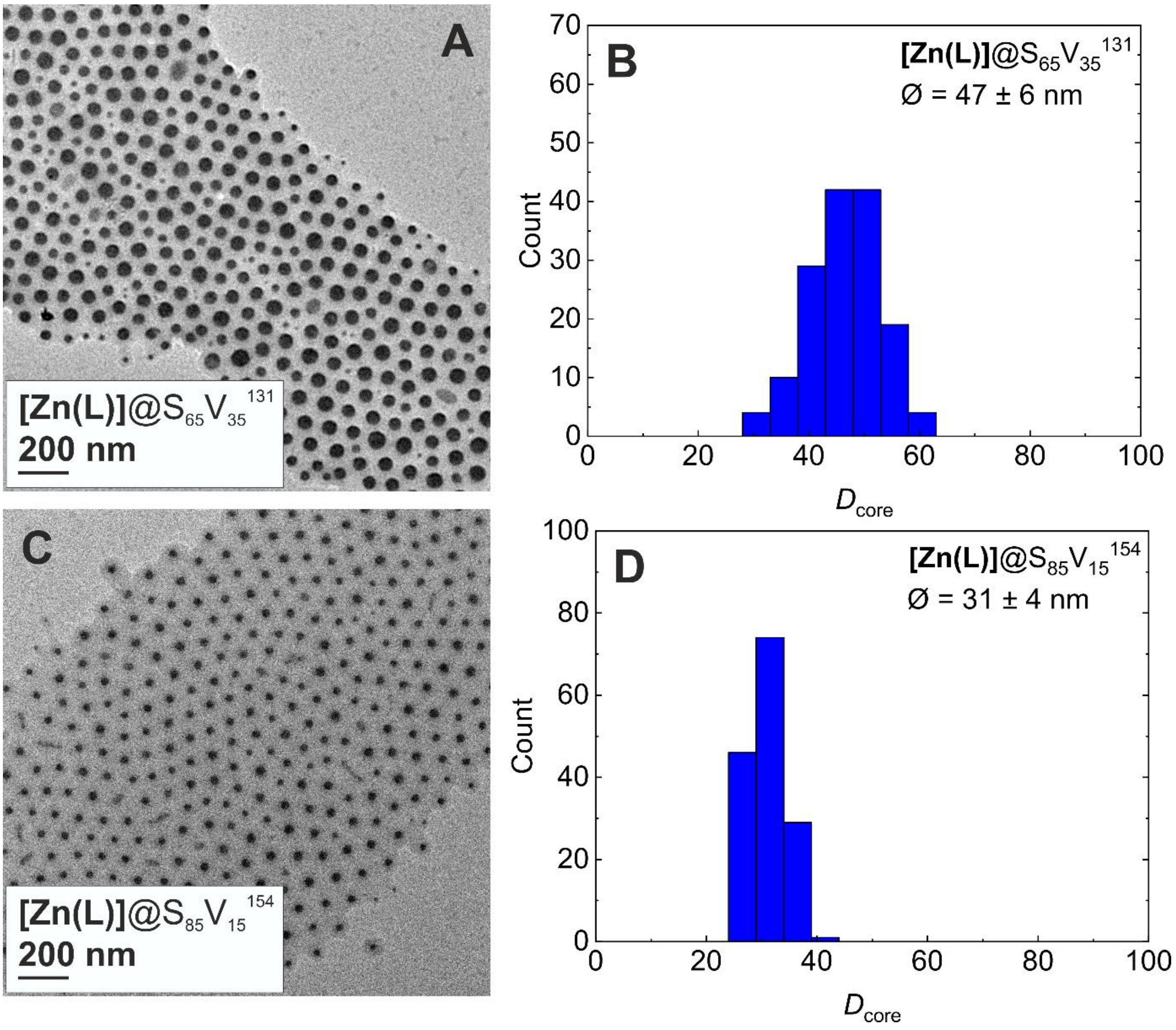

Figure S4: TEM images of **[Zn(L)]**@$S_{65}V_{35}^{131}$ (A) and **[Zn(L)]**@$S_{85}V_{15}^{154}$ (C) micelles in toluene and corresponding core size (D_{core}) distributions derived from TEM image analysis (B, D). Due to the higher electron density contrast the Zn-complex loaded P4VP cores of the micelles appear dark in TEM.

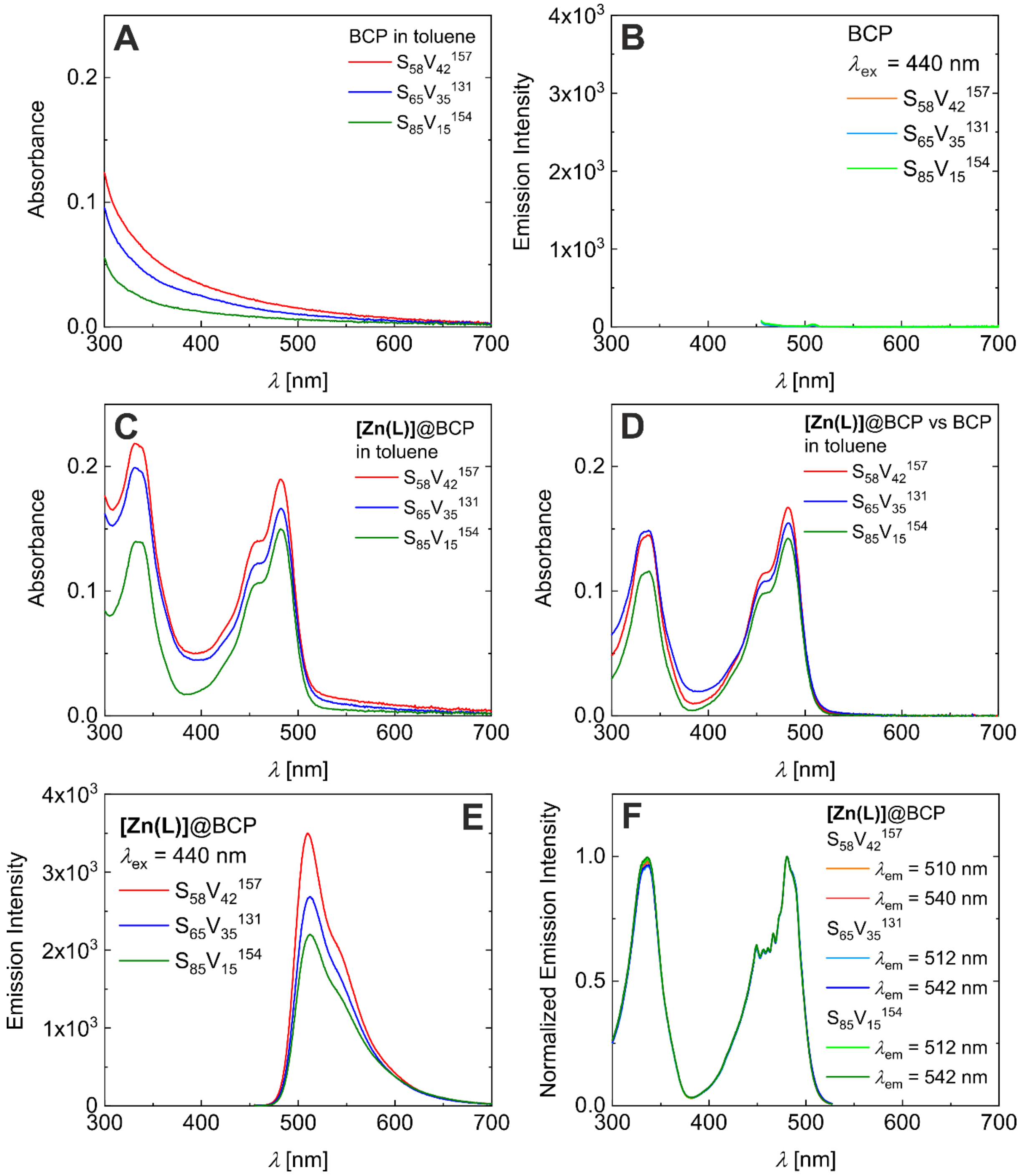

Figure S5: Absorbance (A) and emission (λ_{ex} = 440 nm, B) spectra of neat BCP micelles in toluene. Absorbance spectra of **[Zn(L)]**@BCP micelles in toluene corrected *vs*. neat toluene (C) and vs the neat BCP micelles (D), respectively. Emission spectra (E) and fluorescence excitation spectra (F) of **[Zn(L)]**@BCP micelles in toluene.

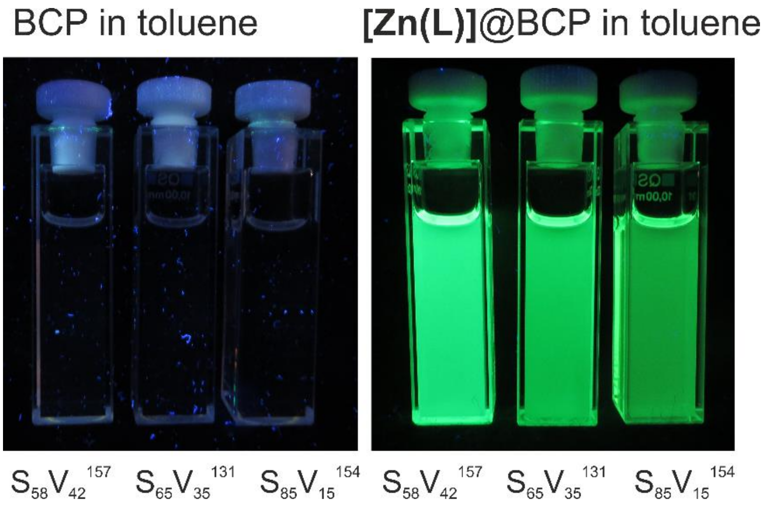

Figure S6: Photograph of the neat BCP micelles (A) and **[Zn(L)]**@BCP micelles (B) in toluene (c = 0.2 g/L) under irradiation with λ_{ex} = 365 nm.

<u>Calculation of equivalents of vinylpyridine compared to **[Zn(L)(H$_2$O)(MeOH)]**</u>

The calculation is exemplary given for the $S_{58}V_{42}^{157}$ BCP.

$$m(P4VP) = 0.42 \cdot 0.004\ g = 1.68 \cdot 10^{-3} g$$

$$n(4VP) = \frac{m(P4VP)}{M(4VP)} = \frac{1.68 \cdot 10^{-3} g}{105.14\ g\ mol^{-1}} = 1.60 \cdot 10^{-5} mol$$

$$n([\mathbf{Zn}(\mathbf{L})(\mathbf{H_2O})(\mathbf{MeOH})]) = \frac{m([\mathbf{Zn}(\mathbf{L})(\mathbf{H_2O})(\mathbf{MeOH})])}{M([\mathbf{Zn}(\mathbf{L})(\mathbf{H_2O})(\mathbf{MeOH})])} = \frac{4 \cdot 10^{-5}\ g}{473.75\ g/mol}$$
$$= 8.44 \cdot 10^{-8}\ mol$$

The equivalents of vinylpyridine relative to **[Zn(L)(H$_2$O)(MeOH)]** can be calculated:

$$Eq = \frac{n(4VP)}{n([\mathbf{Zn}(\mathbf{L})(\mathbf{H_2O})(\mathbf{MeOH})])} = \frac{1.60 \cdot 10^{-5} mol}{8.44 \cdot 10^{-8}\ mol} = 190$$

Table S1: Calculated n(4VP) and equivalents of vinylpyridine relative to **[Zn(L)(H$_2$O)(MeOH)]**.

	n(4VP) [mol]	Equivalents
$S_{58}V_{42}^{157}$	$1.60 \cdot 10^{-5}$	190
$S_{65}V_{35}^{131}$	$1.33 \cdot 10^{-5}$	158
$S_{85}V_{15}^{154}$	$5.71 \cdot 10^{-6}$	68

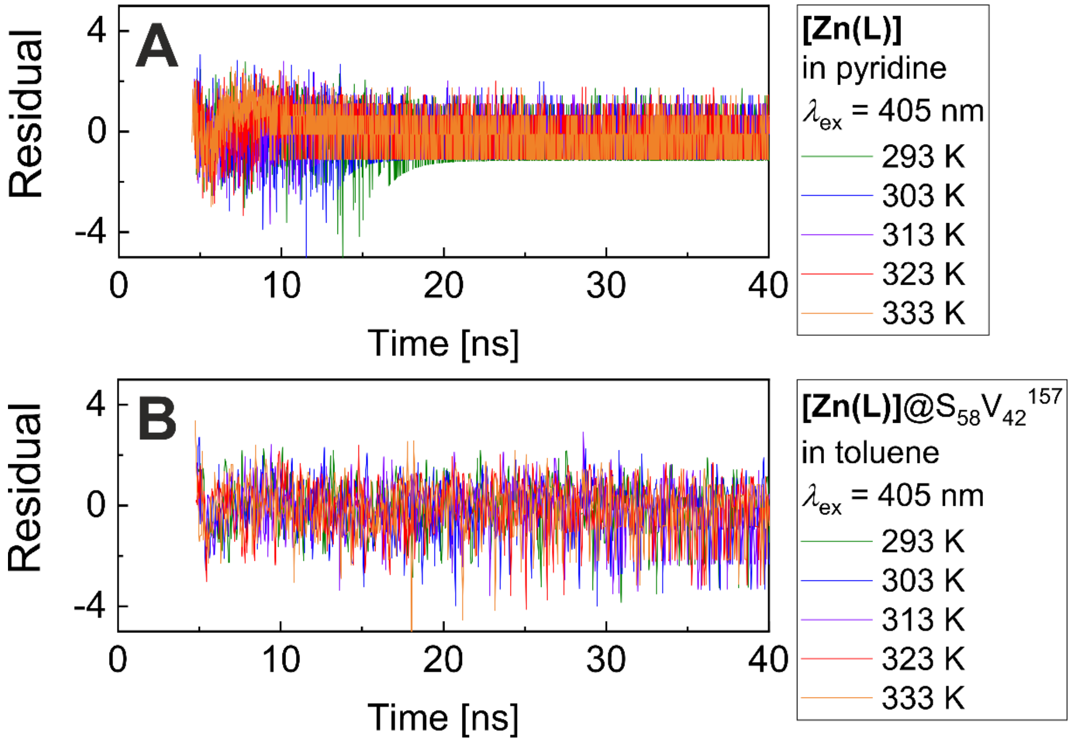

Figure S7: Residuals of the TCSPC detected fluorescence decays of **[Zn(L)]** in pyridine (A) and of **[Zn(L)]**@$S_{58}V_{42}^{157}$ micelles in toluene (B) (λ_{ex} = 405 nm; λ_{em} = 509 nm).

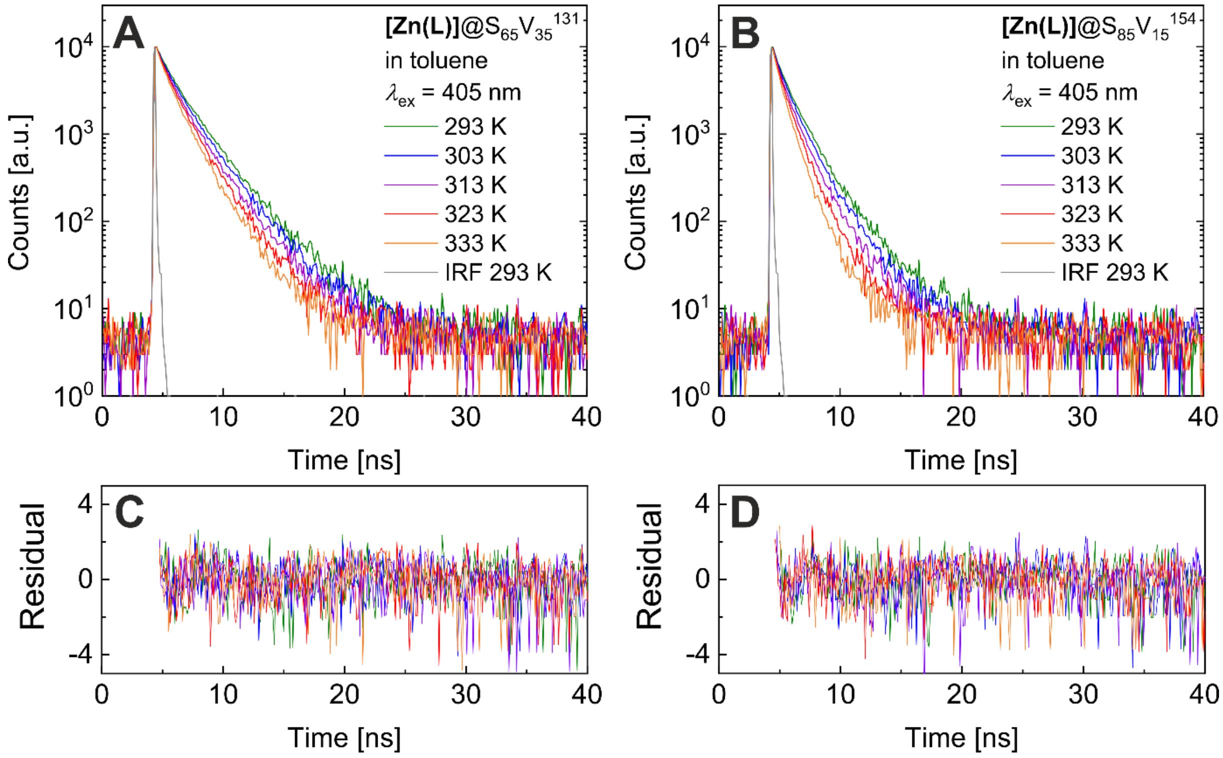

Figure S8: TCSPC detected fluorescence decays of **[Zn(L)]**@$S_{65}V_{35}^{131}$ (A) with residuals (C) and **[Zn(L)]**@$S_{85}V_{15}^{154}$ (B) with residuals (D) in toluene (λ_{ex} = 405 nm; λ_{em} = 509 nm; IRF = instrumental resolution file).

Table S2: Lifetimes τ of **[Zn(L)]** in pyridine and **[Zn(L)]**@BCP in toluene derived from the lifetime measurements at different temperatures.

		293 K	303 K	313 K	323 K	333 K
**[Zn(L)]** in pyridine	τ_1 [ns]	1.319	1.000	0.723	0.500	0.372
**[Zn(L)]**@$S_{58}V_{42}^{157}$ in toluene	τ_1 [ns]	3.020	2.901	2.587	2.530	2.330
	τ_2 [ns]	1.710	1.680	1.415	1.490	1.330
**[Zn(L)]**@$S_{65}V_{35}^{131}$ in toluene	τ_1 [ns]	2.940	2.700	2.433	2.331	2.052
	τ_2 [ns]	1.490	1.357	1.169	1.159	1.009
**[Zn(L)]**@$S_{85}V_{15}^{154}$ in toluene	τ_1 [ns]	2.780	2.380	2.104	1.830	1.970
	τ_2 [ns]	1.236	1.071	0.920	0.837	0.822

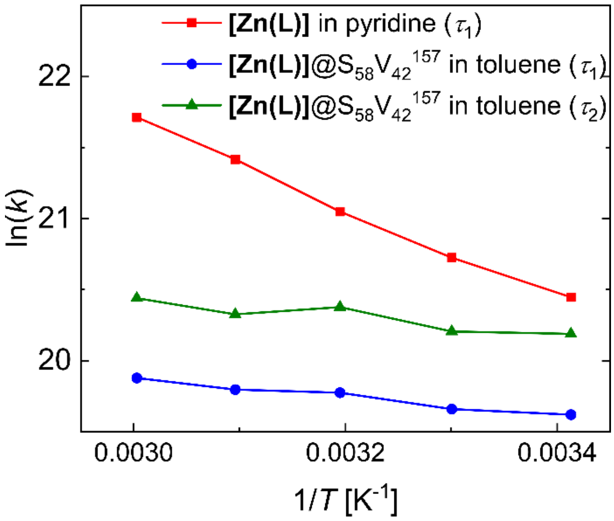

Figure S9: Arrhenius-plot of **[Zn(L)]** in pyridine and **[Zn(L)]**@$S_{58}V_{42}^{157}$ in toluene derived from τ_1 and τ_1.

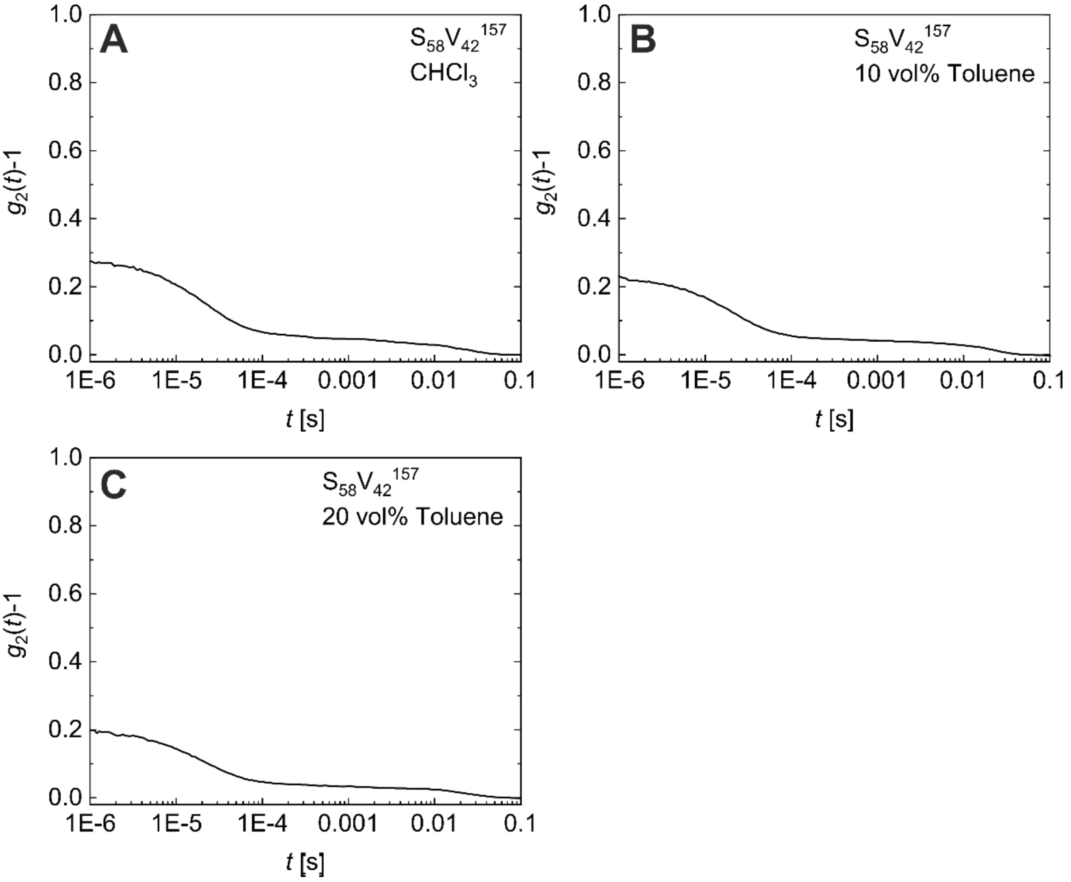

Figure S10: DLS measurements of neat $S_{58}V_{42}^{157}$ in a $CHCl_3$-toluene series: Auto-correlation function $g_2(t)$-1 *vs*. *t* of $S_{58}V_{42}^{157}$: 0 vol% toluene (A), 10 vol% toluene (B), and 20 vol% toluene (C). The D_h distributions were not determined due to the very weak scattering intensities.

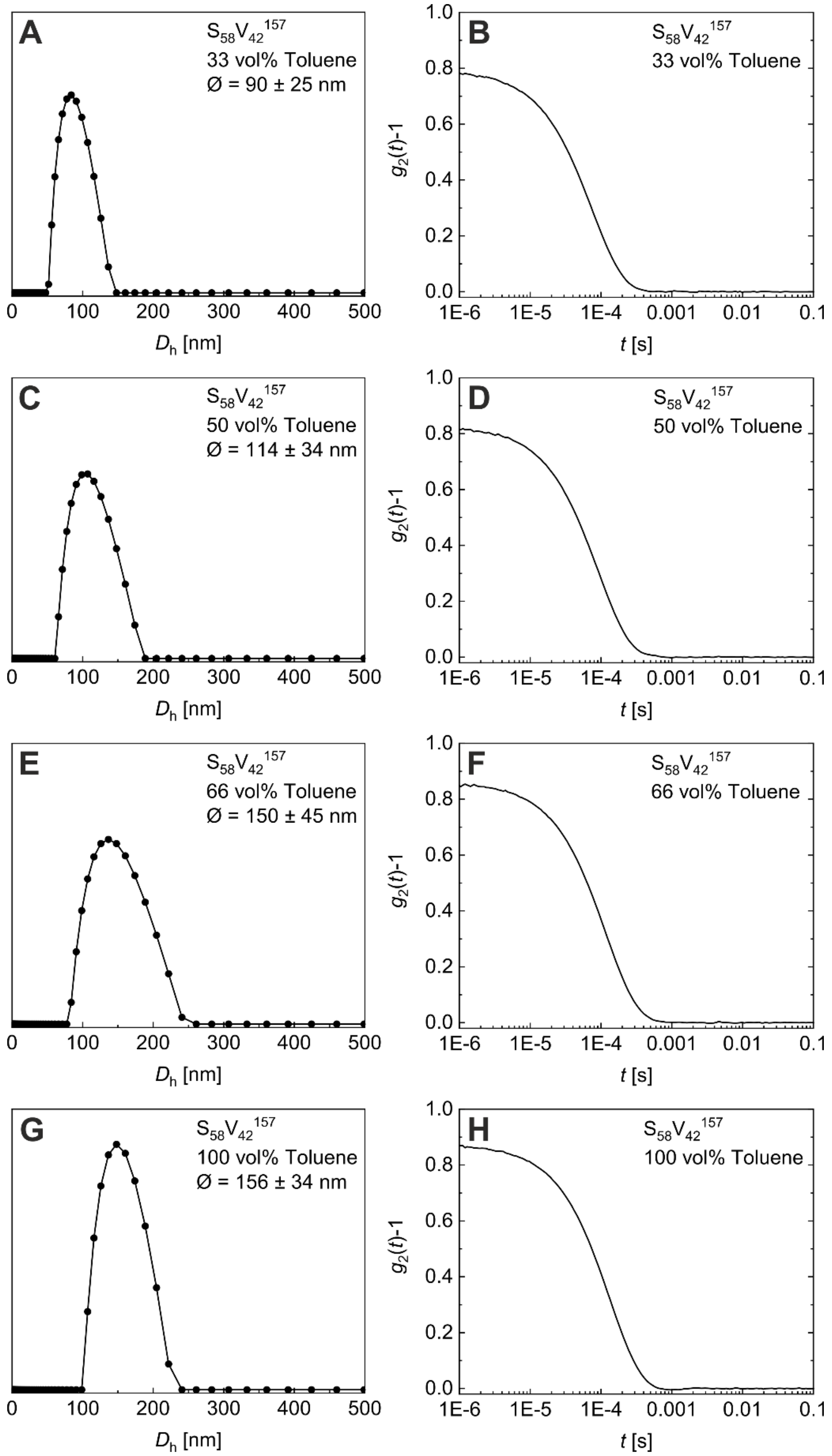

Figure S11: DLS measurements of neat $S_{58}V_{42}^{157}$ micelles in a $CHCl_3$-toluene series: D_h distributions and corresponding autocorrelation functions $g_2(t)$-1 *vs*. t of $S_{58}V_{42}^{157}$: 33 vol% toluene (A/B), 50 vol% toluene (C/D), 66 vol% toluene (E/F), and 100 vol% toluene (G/H).

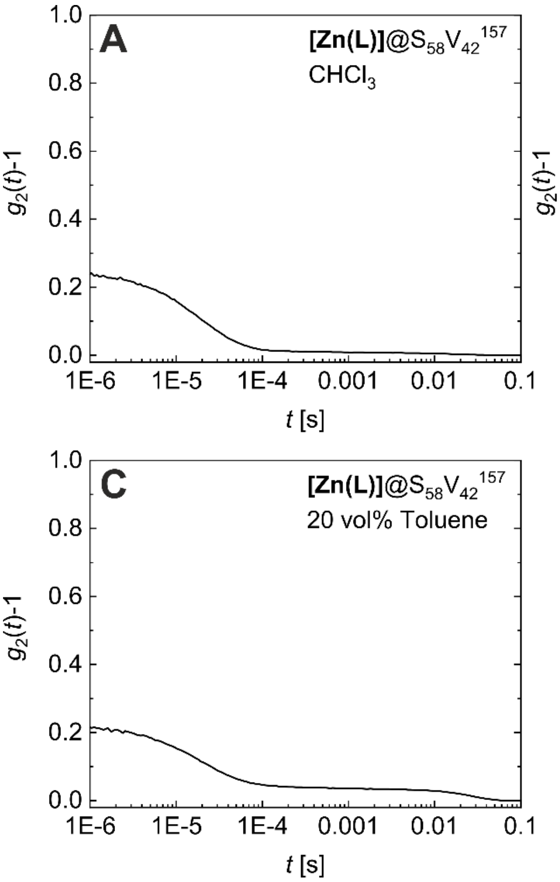

Figure S12: DLS measurements of **[Zn(L)]**@$S_{58}V_{42}{}^{157}$ in a $CHCl_3$-toluene series: Auto-correlation function $g_2(t)$-1 *vs*. *t* of **[Zn(L)]**@$S_{58}V_{42}{}^{157}$: 0 vol% toluene (A), 10 vol% toluene (B), and 20 vol% toluene (C). The D_h distributions were not determined due to the very weak scattering intensities.

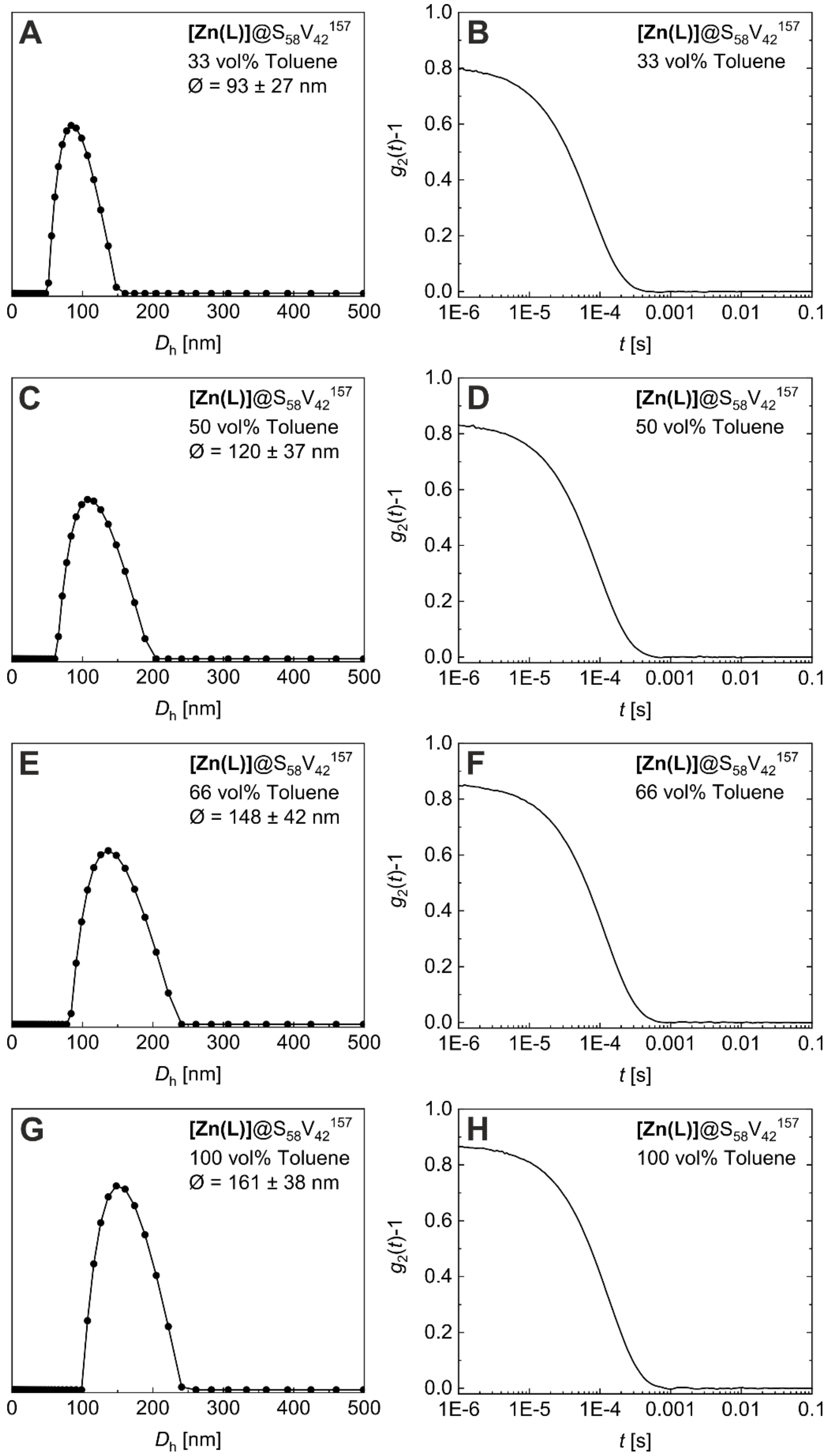

Figure S13: DLS measurements of **[Zn(L)]**@$S_{58}V_{42}^{157}$ micelles in a $CHCl_3$-toluene series: D_h distributions and corresponding autocorrelation functions $g_2(t)$-1 *vs*. *t* of **[Zn(L)]**@$S_{58}V_{42}^{157}$: 33 vol% toluene (A/B), 50 vol% toluene (C/D), 66 vol% toluene (E/F), and 100 vol% toluene (G/H).

Calculation of [$4VP_{eff}$] in the molecularly dissolved ($CHCl_3$) and in the micelle form (toluene)

Exemplarily for **[Zn(L)]**@$S_{58}V_{42}^{157}$:

$$m(4VP) = 0.42 \cdot 0.004\ g = 1.68 \cdot 10^{-3} g$$

$$n(4VP) = \frac{m(4VP)}{M(4VP)} = \frac{1.68 \cdot 10^{-3} g}{105.14\ g\ mol^{-1}} = 1.60 \cdot 10^{-5} mol$$

In the molecularly dissolved form of the BCP, the BCP is solved in 30 mL chloroform.

$$[4VP_{eff,molecularly\ dissolved}] = \frac{n(4VP)}{V} = \frac{1.60 \cdot 10^{-5} mol}{0.03\ L} = 5.33 \cdot 10^{-4} \frac{mol}{L}$$

In the micelle form of the BCP, the poly(4-vinylpyridine) block forms the core of the micelles (diameter 43 nm).

$$V(core) = \frac{4}{3}\pi r^3 = \frac{4}{3}\pi \cdot (21.5 \cdot 10^{-9})^3 = 4.16 \cdot 10^{-20} L$$

$$m(core) = \delta(4VP) \cdot V(core) = 1150 \frac{g}{L} \cdot 4.16 \cdot 10^{-20} L = 4.78 \cdot 10^{-17} g$$

$$n(core) = \frac{m(core)}{M(4VP)} = \frac{4.78 \cdot 10^{-17} g}{105.14\ g\ mol^{-1}} = 4.55 \cdot 10^{-19} mol$$

$$[4VP_{eff,micelle}] = \frac{n(core)}{V(core)} = \frac{4.55 \cdot 10^{-19} mol}{4.16 \cdot 10^{-20} L} = 10.94 \frac{mol}{L}$$

Please note, that the calculated values are based on the following assumptions:

- All polymer chains form micelles
- All pyridine substituents are available for ligation

For this reason, the exact values are not determinable. However, the order of magnitude shows that the local pyridine concentration inside the micelles is strongly increased compared to the molecularly dissolved form.

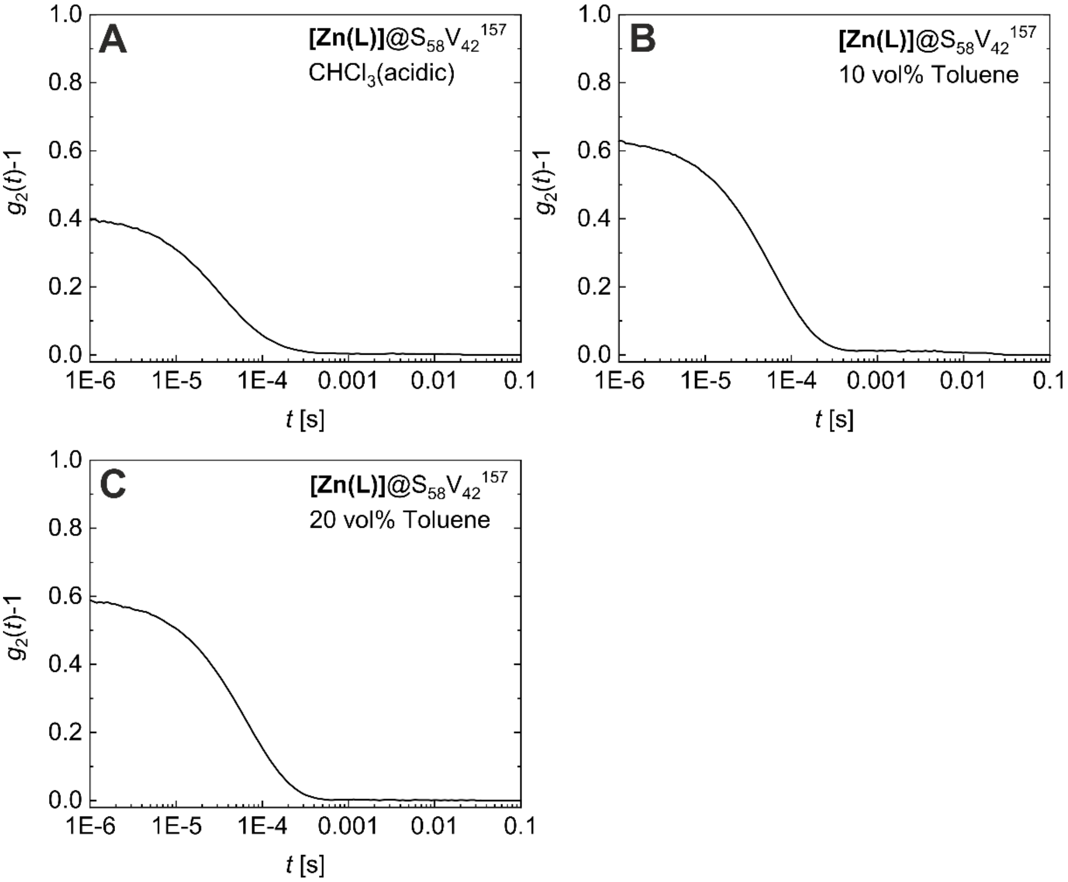

Figure S14: DLS measurements of **[Zn(L)]**@$S_{58}V_{42}^{157}$ in a $CHCl_3$(acidic)-toluene series: Autocorrelation function $g_2(t)$-1 *vs.* *t* of **[Zn(L)]**@$S_{58}V_{42}^{157}$: 0 vol% toluene (A), 10 vol% toluene (B), and 20 vol% toluene (C). The D_h distributions were not determined due to the very weak scattering intensities.

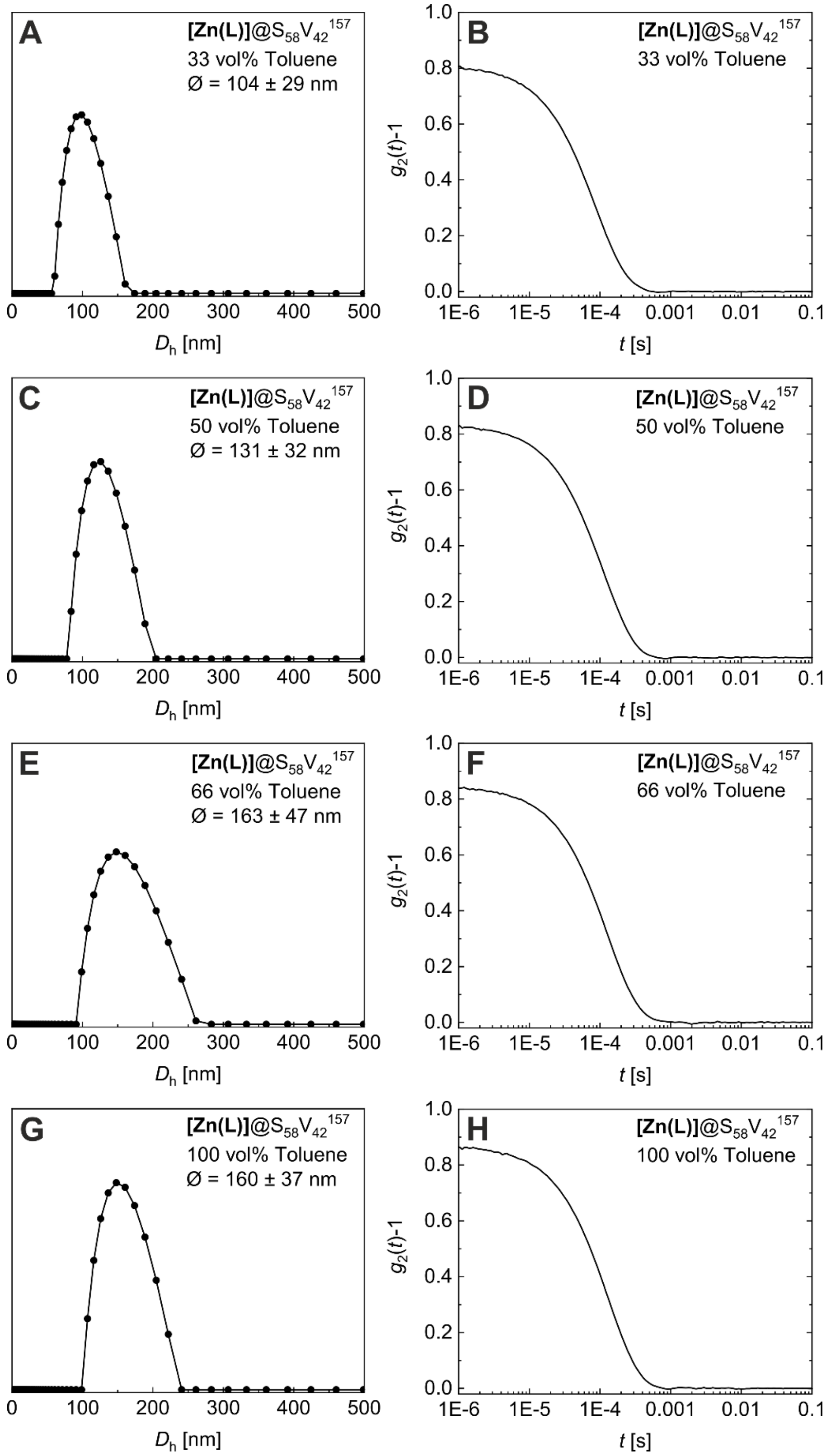

Figure S15: DLS measurements of **[Zn(L)]**@$S_{58}V_{42}^{157}$ micelles in a $CHCl_3$(acidic)-toluene series: D_h distributions and corresponding autocorrelation functions $g_2(t)$-1 *vs.* *t* of **[Zn(L)]**@$S_{58}V_{42}^{157}$: 33 vol% toluene (A/B), 50 vol% toluene (C/D), 66 vol% toluene (E/F), and 100 vol% toluene (G/H).

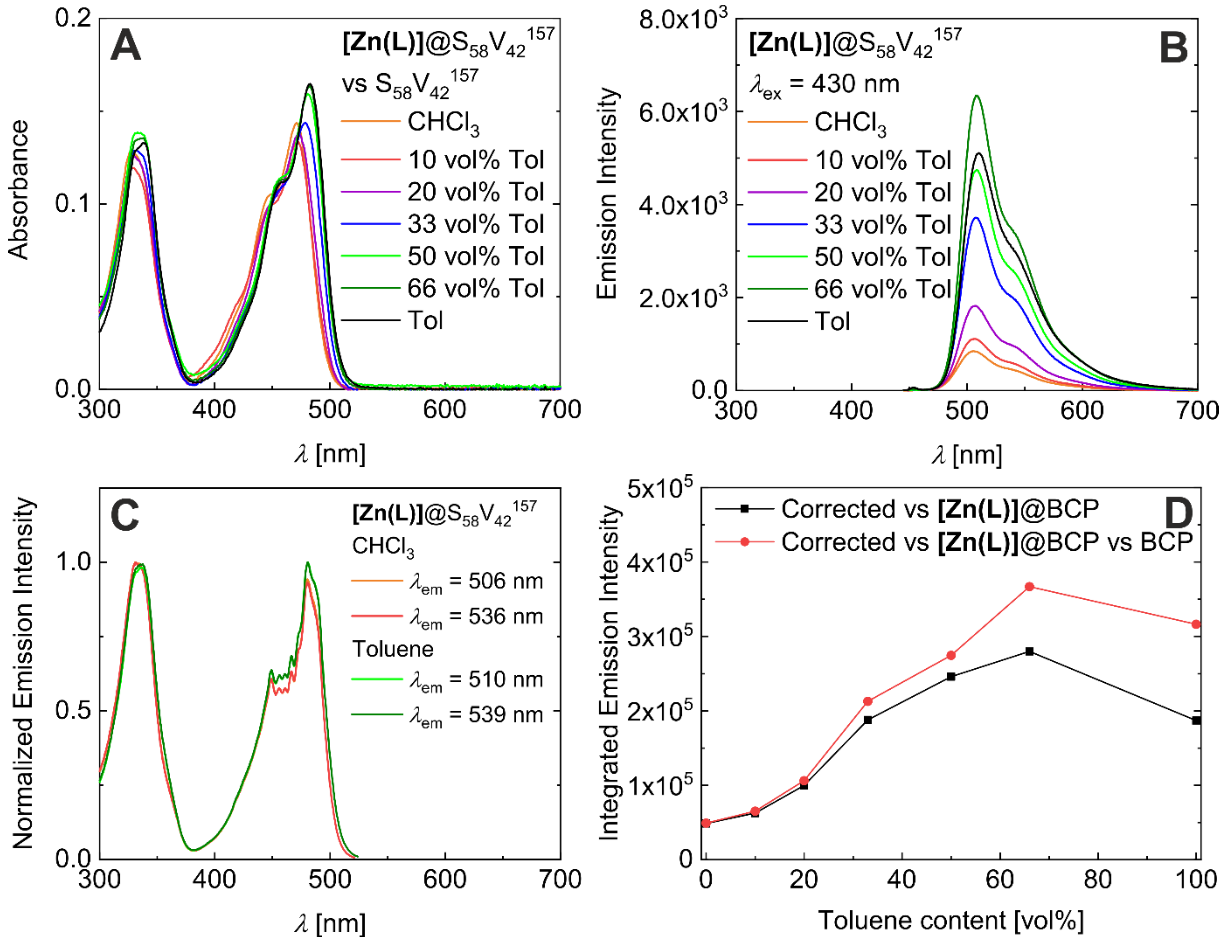

Figure S16: Absorbance and emission spectra of **[Zn(L)]**@$S_{58}V_{42}^{157}$ in a $CHCl_3$-toluene series (A/B; λ_{ex} = 430 nm). Fluorescence excitation spectra of **[Zn(L)]**@$S_{58}V_{42}^{157}$ in $CHCl_3$ and toluene (C). Plot of the integrated emission intensity vs. toluene content of **[Zn(L)]**@$S_{58}V_{42}^{157}$ (D). Both correction possibilities are plotted to show that this trend is retained for both possibilities: The correction of the emission *vs*. the absorbance value at λ_{ex} = 430 nm using the pure solvent as background (red) and using the neat BCP micelles as background (black). The weak emission at approximately λ = 450 nm is due to a measurement artefact

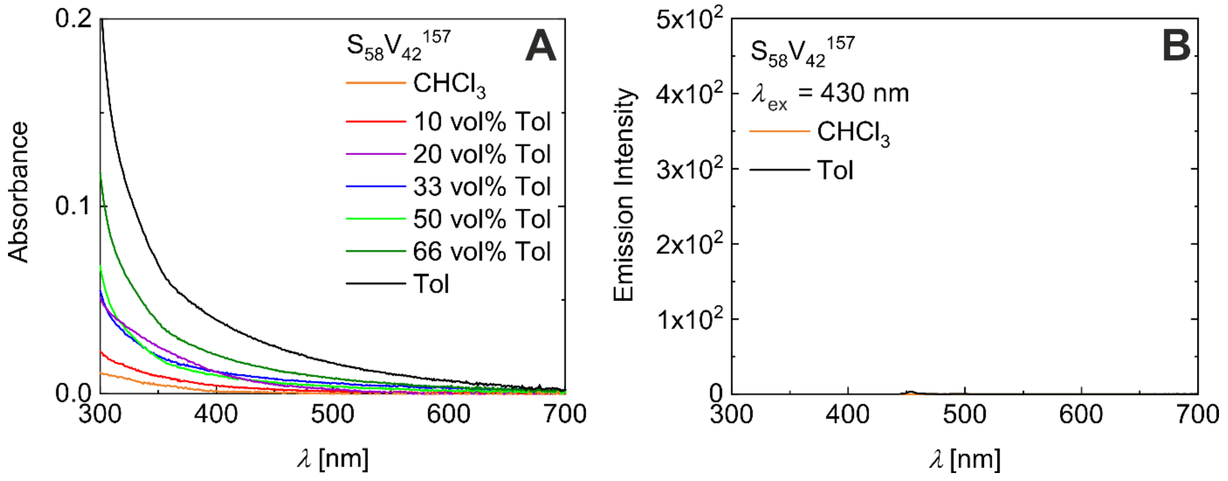

Figure S17: Absorbance spectra of $S_{58}V_{42}^{157}$ in a $CHCl_3$-toluene series (A). Emission spectra of $S_{58}V_{42}^{157}$ in $CHCl_3$ and toluene (λ_{ex} = 430 nm, B). The weak emission at approximately λ = 450 nm is due to a measurement artefact

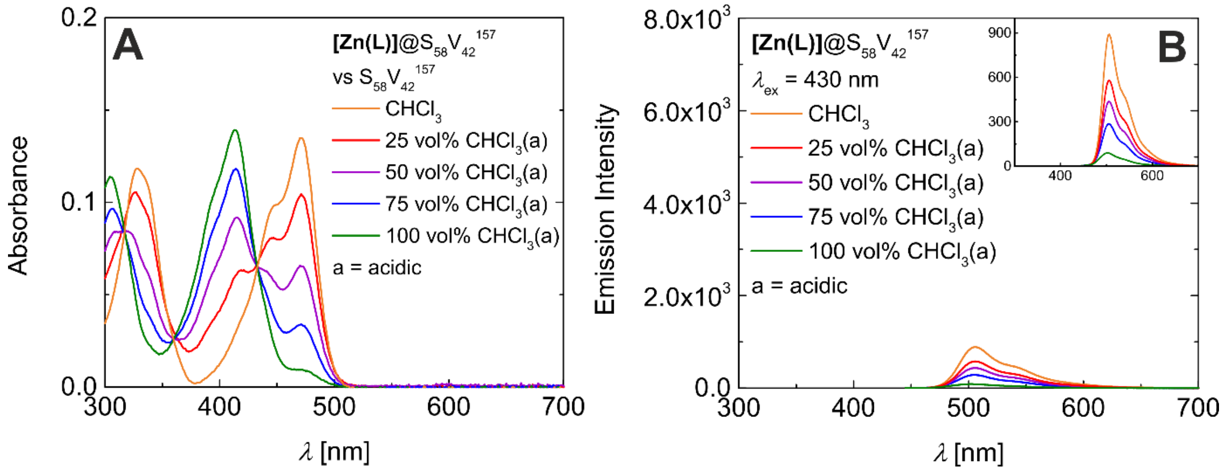

Figure S18: Absorbance and emission spectra of **[Zn(L)]**@$S_{58}V_{42}^{157}$ in a $CHCl_3$-$CHCl_3$(acidic) series (A/B; λ_{ex} = 430 nm). The weak emission at approximately λ = 450 nm is due to a measurement artefact

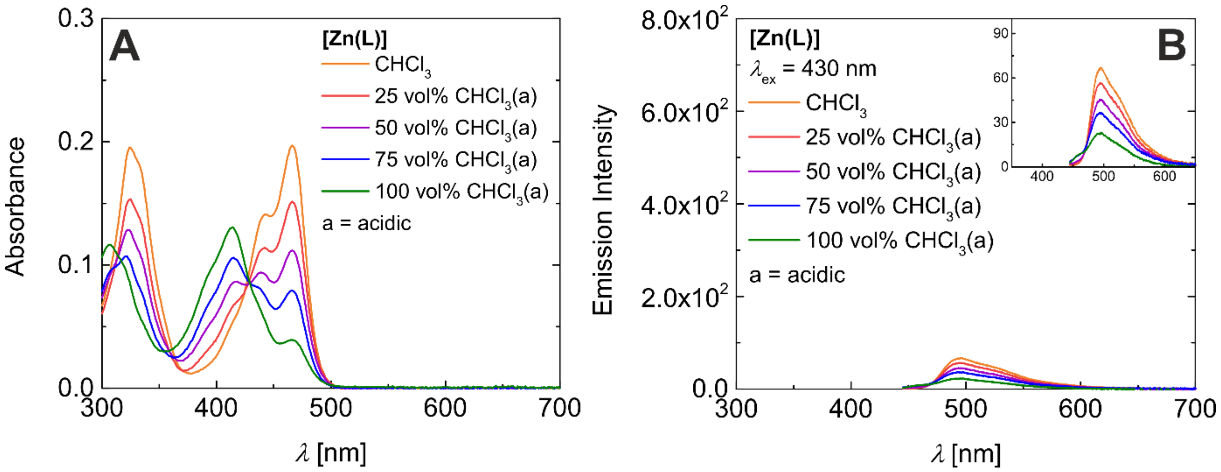

Figure S19: Absorbance and emission spectra of **[Zn(L)]** in a $CHCl_3$-$CHCl_3$(acidic) series (A/B; λ_{ex} = 430 nm). The weak emission at approximately λ = 450 nm is due to a measurement artefact

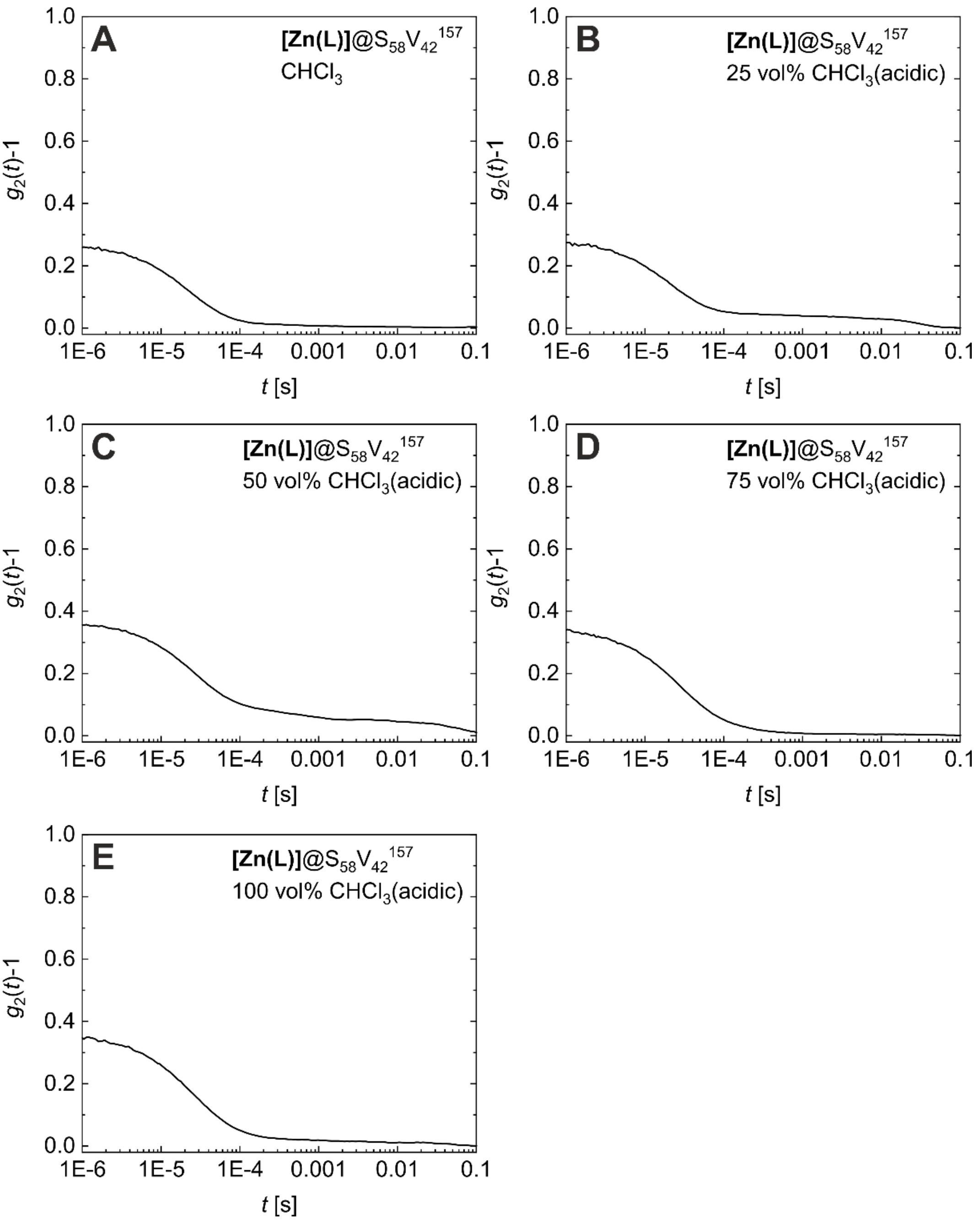

Figure S20: DLS measurements of **[Zn(L)]**@$S_{58}V_{42}^{157}$ in a $CHCl_3$-$CHCl_3$(acidic) series: Autocorrelation function $g_2(t)$-1 *vs*. *t* of **[Zn(L)]**@$S_{58}V_{42}^{157}$ in $CHCl_3$ (A), 25 vol% $CHCl_3$(acidic) (B), 50 vol% $CHCl_3$(acidic) (C), 75 vol% $CHCl_3$(acidic) (D), and 100 vol% $CHCl_3$(acidic) (E). The D_h distributions were not determined due to the very weak scattering intensities.

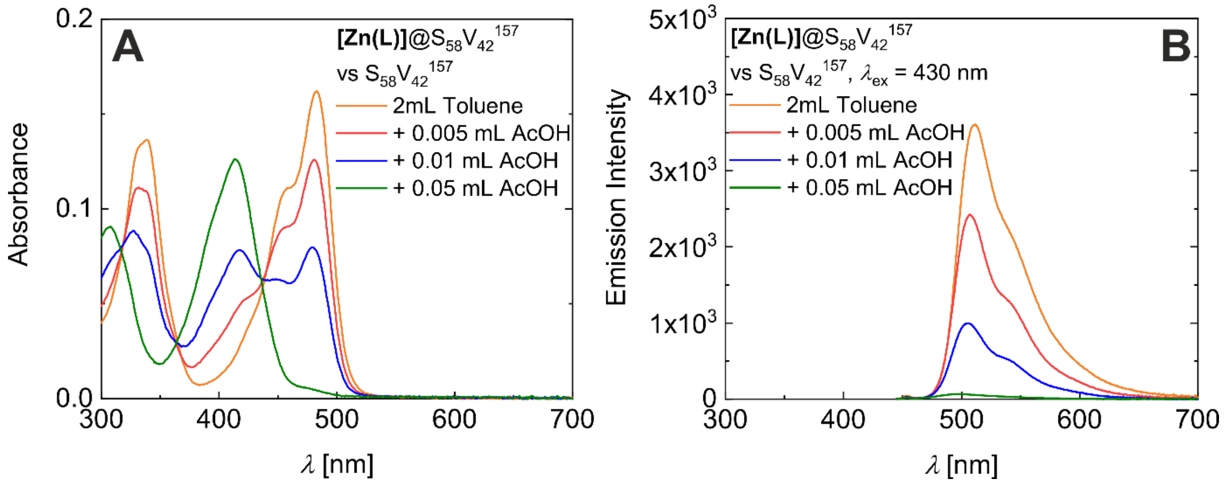

Figure S21: Absorbance (A) and emission (B, λ_{ex} = 430 nm) spectra of **[Zn(L)]**@ $S_{58}V_{42}^{157}$ in toluene in dependence of the amount of added acetic acid. The weak emission at approximately λ = 450 nm is due to a measurement artefact

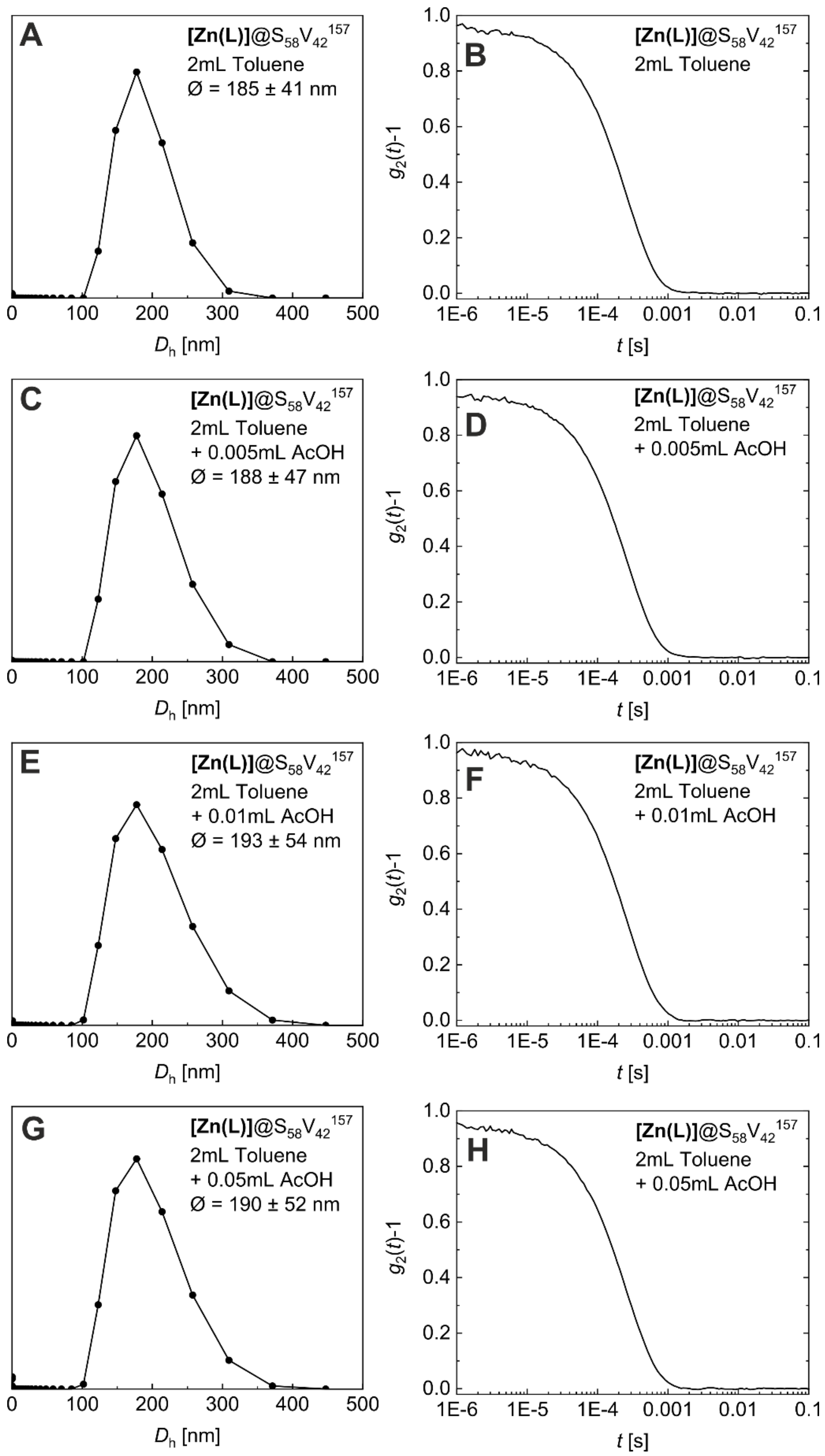

Figure S22: DLS measurements of **[Zn(L)]**@$S_{58}V_{42}^{157}$ micelles in toluene in dependence of the amount of added acetic acid: D_h distributions and corresponding autocorrelation functions $g_2(t)$-1 *vs*. *t* of **[Zn(L)]**@$S_{58}V_{42}^{157}$: toluene (A/B), 2mL toluene + 0.005mL AcOH (C/D), 2mL toluene + 0.01mL AcOH (E/F), and 2mL toluene + 0.05mL AcOH (G/H). The measurements were performed on a different instrument.

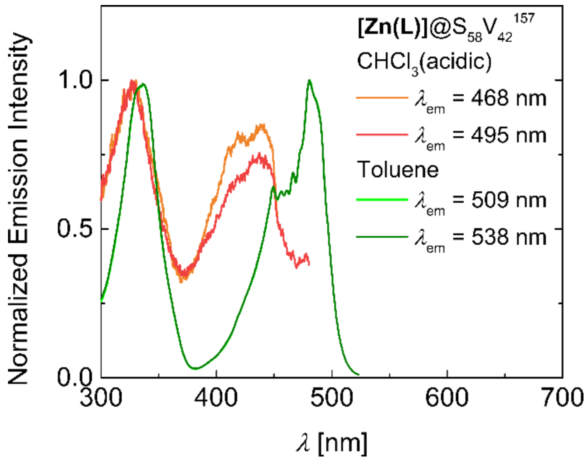

Figure S23: Normalized fluorescence excitation spectra of **[Zn(L)]**@$S_{58}V_{42}$[157] in toluene and $CHCl_3$(acidic).

[1] H. Kurz, G. Hörner, O. Weser, G. L. Manni, B. Weber, *Chem. Eur. J.* **2021**, *27*, 15159.

[2] a) C. Göbel, G. Hörner, A. Greiner, H. Schmalz, B. Weber, *Nanoscale Adv.* **2020**, *2*, 4557; b) C. Göbel, C. Hils, M. Drechsler, D. Baabe, A. Greiner, H. Schmalz, B. Weber, *Angew. Chem. Int. Ed.* **2020**, *59*, 5765; c) C. Göbel, C. Hils, M. Drechsler, D. Baabe, A. Greiner, H. Schmalz, B. Weber, *Angew. Chem.* **2020**, *132*, 5814.

[3] C. Göbel, K. Marquardt, D. Baabe, M. Drechsler, P. Loch, J. Breu, A. Greiner, H. Schmalz, B. Weber, *DOI: 10.26434/chemrxiv-2021-jbj2n*, **2021**.

[4] W. S. Rasband, *ImageJ Bethesda, National Institute of Health, Maryland, USA*, **1997**.
